# Apoptotic dysregulation mediates stem cell competition and tissue regeneration

**DOI:** 10.1038/s41467-023-41684-x

**Published:** 2023-11-20

**Authors:** Marianna Yusupova, Roi Ankawa, Yahav Yosefzon, David Meiri, Ido Bachelet, Yaron Fuchs

**Affiliations:** 1 Faculty of Biology, Technion-Israel Institute of Technology, Haifa, Israel; 2 Augmanity, Rehovot, Israel

**Keywords:** Skin stem cells, Regeneration, Stem-cell niche

## Abstract

Since adult stem cells are responsible for replenishing tissues throughout life, it is vital to understand how failure to undergo apoptosis can dictate stem cell behavior both intrinsically and non-autonomously. Here, we report that depletion of pro-apoptotic Bax protein bestows hair follicle stem cells with the capacity to eliminate viable neighboring cells by sequestration of TNFα in their membrane. This in turn induces apoptosis in “loser” cells in a contact-dependent manner. Examining the underlying mechanism, we find that Bax loss-of-function competitive phenotype is mediated by the intrinsic activation of NF*κ*B. Notably, winner stem cells differentially respond to TNFα, owing to their elevated expression of TNFR2. Finally, we report that in vivo depletion of Bax results in an increased stem cell pool, accelerating wound-repair and de novo hair follicle regeneration. Collectively, we establish a mechanism of mammalian cell competition, which can have broad therapeutic implications for tissue regeneration and tumorigenesis.

## Abstract (web summary)

The authors show that depletion of pro-apoptotic Bax protein bestows hair follicle stem cells with the capacity to eliminate viable neighboring cells by sequestration of TNFα, resulting in an increased stem cell pool, accelerated wound repair and de novo hair follicle regeneration.

## Introduction

Cell competition is a process that was originally described in the *drosophila* imaginal disc, in which wild-type (WT) cells of greater fitness were capable of sensing and inducing the death of lesser-fit mutant cells^1^. Since its discovery, it has been established as a critical regulator of homeostasis in a variety of cellular contexts^2–7^. As stem cells (SCs) are the cellular drivers of organismal development and tissue replenishment, it is unsurprising that they rely on cell competition as a quality control mechanism to eliminate unfit SCs from the tissues in which they reside^8^. This process is therefore intimately coupled with the ability of SCs to undergo programmed cell death (PCD), with previous studies having demonstrated the importance of apoptosis in SC-mediated homeostasis and tissue regeneration^9–13^.

A critical point along the apoptotic cascade is the oligomerization of the pro-apoptotic Bax and Bak proteins to create pores in the mitochondria, resulting in mitochondrial outer membrane permeabilization (MOMP)^11^. MOMP in turn culminates in the activation of caspases that cleave a variety of vital substrates and implement the cell death program^14^. Activation of Bax and Bak has therefore been considered as a “point of no return” in apoptotic execution and represents a critical upstream event for apoptotic intervention^14–18^.

To investigate the consequences of apoptotic dysregulation on SC-dependent processes, we harnessed hair follicle stem cells (HFSCs), which have an essential and extensively characterized role in tissue homeostasis and repair. Additionally, previous studies have demonstrated the importance of apoptosis in regulating these cells and their dependent processes^9,12^. Our data show that targeting of Bax not only conferred HFSCs with apoptotic resistance and enhanced proliferative capabilities, but also rendered them as super competitors capable of killing otherwise healthy neighboring cells. Exploiting TNFα, Bax-depleted cells induced apoptosis of WT neighbors in a contact-dependent manner and evaded elimination. Finally, mice depleted for Bax displayed HF hypertrophy, as well as accelerated wound repair and de novo HF regeneration post-wounding. Taken together, we reveal critical consequences for Bax-mediated apoptotic dysregulation in triggering competitive cell elimination and altering tissue homeostasis and regeneration.

## Results

### Bax regulates apoptotic resistance and proliferation

**Fig. 1:**
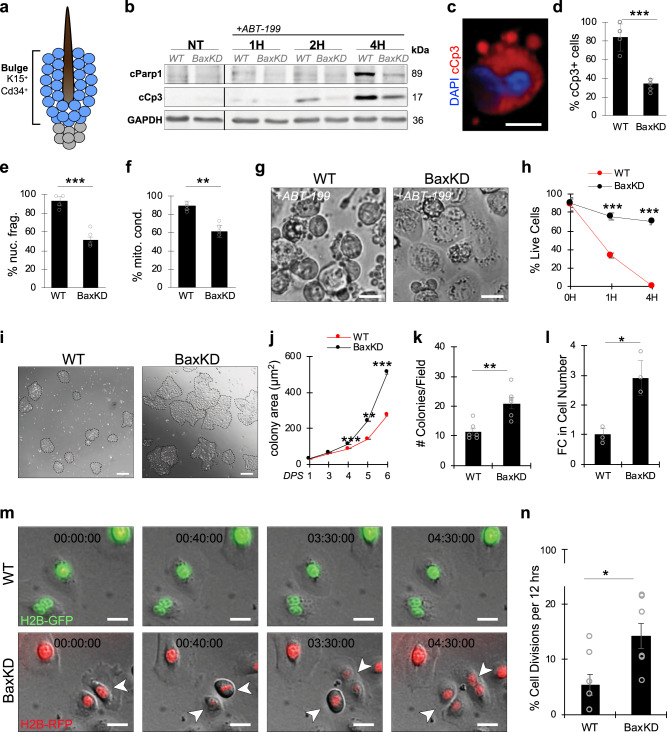
Bax depletion confers enhanced apoptotic resistance and proliferation. **a** Schematic depiction of the K15^+^CD34^+^ stem cell (SC) population in the hair follicle (HF) bulge. **b** Immunoblotting (IB) of wild-type (WT) or Bax knockdown (BaxKD) HFSCs treated with apoptotic-inducing ABT199 against cleaved Parp1 (cParp1), cleaved Caspase-3 (cCp3) and GAPDH. **c** Immunofluorescent (IF) staining against cCp3 representing apoptotic cell morphology (derived from Supplementary Fig. 1d (bottom panel) of an ABT-199 treated BaxKD cell). **d** Percentage of cCp3^+^ in WT and BaxKD HFSCs after 4 h of ABT199 treatment. **e** Percentage of cells with nuclear fragmentation after ABT199 treatment based on (**c**). *n* = 4 biological replicates examined per condition. **f** Percentage of cells with mitochondrial condensation based on Supplementary Fig. 1e. **g** Brightfield (BF) images of WT or BaxKD cells treated with ABT199 after 4 h. **h** Percentage of cell survival based on (**g**). **i** BF images depicting WT or BaxKD HFSC colonies after 6 days post-seeding (DPS). **j** Quantification of colony growth between 1 and 6 DPS based on (**i**). A minimum of 75 colonies across *n* = 12 randomly selected fields and *n* = 3 biological replicates examined. **k** Quantification of number of colonies per field after 6DPS based on (***i***). *n* = 6 randomly selected fields across *n* = 3 biological replicates examined per condition. **l** Fold change (FC) in cell number per colony after 6DPS based on (***i***). A minimum of 1,400 cells across 3 fields and n = 3 biological replicates examined per condition. **m** Live BF and fluorescent imaging of WT or BaxKD HFSCs across 4.5 h. Arrowheads denote cells undergoing mitosis. **n** Percentage of WT or BaxKD HFSCs undergoing cell division within 12 h based on (***m***). Scale bar represents 10 μm for **c**; 20 μm for **g**, **m**; 100 μm for **i**. *n* = 3 biological replicates per condition per experiment unless otherwise specified. Source data are provided as a Source Data file. Each experiment was repeated at least 2 times with similar results. All data are mean and ± SEM. *P* values were determined by unpaired two-tailed t-test. *P* value < 0.05*, <0.01**, <0.001***.

Utilizing single-cell RNA sequencing (SC RNA-seq) data of the telogenic mouse epidermis^19^, we evaluated the expression of these proteins in the HFSC bulge (CD34^+^K15^+^) populations^20,21^ (Fig. 1a) known to harness apoptosis for homeostasis, and found that Bax and not Bak1 was predominantly expressed in this subset of SCs (Supplementary Fig. 1a) (GSE142471). Although Bax and Bak1 have been described to display some level of redundancy^17,18,22^, several studies have demonstrated distinct non-redundant roles^15,23,24^. Additionally, a mutation in Bax has specifically been implicated in various disease pathologies, including squamous cell carcinoma and intestinal cancer^25–27^. We therefore decided to target Bax to assess whether this would be sufficient to dysregulate apoptosis and confer HFSCs with apoptotic resistance. Towards this, we isolated α6^+^CD34^+^ScaI^-^ HFSCs^28,29^ and generated lentiviral particles expressing an shRNA under the U6 promoter against a nonsense scrambled (SCR) sequence (henceforth referred to as WT) or Bax coding sequence (Table S1), thereby enabling stable knockdown of Bax (henceforth referred to as BaxKD). Using quantitative PCR (qPCR), Bax mRNA levels were found to be reduced by ~80% relative to SCR control HFSCs (Supplementary Fig. 1b). This was additionally verified on a protein level via immunostaining against Bax (Supplementary Fig. 1c). Next, BaxKD or WT cells were treated with the pro-apoptotic compound ABT199, a potent Bcl-2 inhibitor, and assessed for degrees of apoptotic induction. Immunoblotting (IB) against apoptotic markers cleaved Caspase-3 (cCp3) and cleaved Parp1 (cParp1) revealed a strong difference in the elevation of these markers in WT versus BaxKD with increasing duration of treatment (Fig. 1b). In verification, immunofluorescent (IF) staining indicated a noticeably greater degree of cCp3^+^ apoptotic cells accompanied by nuclear condensation in WT but not BaxKD HFSCs 4 hours (h) post treatment (Fig. 1c-e; Supplementary Fig. 1d). As additional morphological hallmarks of apoptosis include mitochondrial condensation and membrane blebbing, we utilized the live cell mitochondrial dye (MitoTrackr) to track changes in mitochondrial morphology together with brightfield (BF) imaging to evaluate membrane blebbing. After 4 h of ABT199 treatment, nearly all WT but not BaxKD HFSCs exhibited mitochondrial condensation (Fig. 1f; Supplementary Fig. 1e) as well as a greater degree of membrane blebbing (Fig. 1g), suggesting differential resistance to apoptosis. These findings were also verified via quantification of cell survival via Trypan Blue staining, which demonstrated a significant decrease in the percentage of surviving WT compared to BaxKD HFSCs (Fig. 1h). Finally, induction of apoptosis via combined treatment with the death ligand TNFα and protein synthesis inhibitor cycloheximide (CHX) also resulted in increased membrane blebbing and decreased survival of WT compared with BaxKD HFSCs (Supplementary Fig. 1f-g). These data are consistent with previous findings, which demonstrated that cells deficient in Bax or Bak exhibited apoptotic resistance upon withdrawal of growth promoting factors, as well as in the presence of various apoptotic stimuli^16,22,30^.

Notably, we observed that a distinct feature of the BaxKD HFSCs in culture was an increased expansion potential. We found that in contrast to control HFSCs, BaxKD cells generated approximately two-fold more colonies, which were on average nearly twice as large 6DPS (Fig. 1i-k). In accordance, the number of BaxKD HFSCs was increased by approximately three-fold (Fig. 1l). Although this could potentially be explained by conferred resistance to apoptosis, we hypothesized that the dramatic expansion could at least partially be a result of affected cell proliferation. Therefore, we performed time-lapse microscopy of fluorescently labeled WT and BaxKD HFSCs, which indicated that ~2.6-fold more BaxKD HFSCs underwent cell divisions in a 12-hour period (Fig. 1m, n). However, while we observed an increase in the number of mitotic events, no effect was seen on the duration of the cell cycle (Supplementary Fig. 1h). Consistent with this, IF staining against proliferative marker Ki-67 also revealed an increased percentage of Ki-67^+^ cells in BaxKD versus WT cultures (Supplementary Fig. 1i, j). These findings indicate that Bax depletion affects both apoptotic resistance as well as HFSC proliferation, thus increasing the fitness of these cells.

### Apoptotic dysregulation promotes cell competition

Previously, imbalances in cellular fitness have been shown to encourage selective clonal expansion and in certain cases, provoke competition between cell populations residing within a shared environment^1–7^. Furthermore, mutations and other changes conferring enhanced cellular fitness were discovered to promote the super competitive elimination of otherwise viable WT cells^5,6^. Such changes include but are not limited to enhanced proliferation, protein synthesis, sensitivity to pro-survival signals, and the ability to secrete death ligands and other antagonistic signals to neighboring cells within the environment^1,31–33^.

**Fig. 2:**
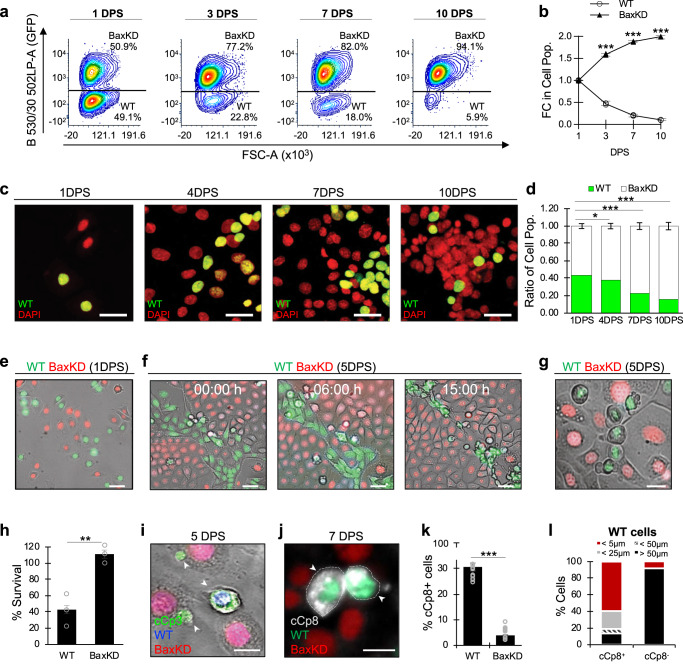
Bax-depleted cells actively eliminate WT neighbors through contact-dependent apoptotic induction. **a** Fluorescent activated cell sorting (FACS) plots depicting co-cultured WT and BaxKD cell populations 1, 3, 7, and 10 days post-seeding (DPS). *n* = 3 biological repeats and representative of 6 experimental repeats. **b** Fold change (FC) in cell populations based on (**a**). **c** Fluorescent images of WT (green, DAPI) and BaxKD (DAPI-only) co-cultures 1, 4, 7, and 10 DPS. **d** Quantification of ratio of WT and BaxKD cell populations from in situ analysis of (**c**). *n* = 3 biological repeats representative of 3 experimental repeats. **e** Live BF and fluorescent imaging of WT (green) and BaxKD (red) co-cultures 1DPS. **f** Live BF and fluorescent imaging of WT and BaxKD co-cultures 5DPS at 0, 6, and 15 h timepoints (0 h represents start of imaging; dashed lines mark population boundaries). **g** Live BF and fluorescent imaging close-up of WT and BaxKD co-cultures. **h** Percentage of cell survival over time based on (**f**). *n* = 3 biological replicates representing more than 600 cells evaluated. **i** IF against cCp3 in WT and BaxKD co-cultures 5DPS. Arrowheads denote apoptotic WT cells. **j** IF against cCp8 in WT and BaxKD co-cultures 7DPS. Arrowheads denote apoptotic WT cells. **k** Percentage of cCp8^+^ cells based on (**j**). *n* = 3 biological replicates with 3 fields containing at least 100 cells examined in each. **l** Percentage of cCp8^+^ or cCp8^-^ WT-RFP^+^ cells in proximity to a BaxKD-GFP^+^ cell in co-cultures based on (**j**). Scale bar represents 25 μm in **c**; 50 μm in **e**, **f**; 20 μm in **g, i**, **j**. *n* = 3 biological replicates per condition per experiment unless otherwise specified. Source data are provided as a Source Data file. Each experiment was repeated at least 2 times with similar results. All data are mean and ± SEM. *P* values were determined by unpaired two-tailed t-test. *P* value < 0.05*, <0.01**, <0.001***.

As we have observed that Bax depletion enhances apoptotic resistance and proliferation, thereby representing an altered cellular fitness state, we wanted to understand whether Bax-depleted SCs cause non-cell autonomous changes in surrounding cells. Towards this, we designed co-culture assays in which fluorescently labeled BaxKD and WT HFSCs were seeded at a 1:1 ratio and changes in each cell population were quantified over time via fluorescent activated cell sorting (FACS). Notably, within 10 days post seeding (10DPS), WT HFSCs cells were depleted from ~50% to ~5% of the total cell population (Fig. 2a, b). In comparison, control cultures of only WT or BaxKD cells displayed no significant changes in the overall ratio of populations (Supplementary Fig. 2a, b). For additional verification of our FACS quantification method, we performed in situ image analysis of co-cultures and observed the same trends of cell depletion (Fig. 2c, d), together indicating that depletion of Bax confers a strong fitness advantage that propels mutant cells to preferentially expand within the population. Although the phenotype could be in part due to enhanced proliferation and accumulation resulting from apoptotic resistance, the degree to which WT cells were depleted by the conclusion of our co-culture experiments suggested that BaxKD cells may actively affect the expansion of control WT cells.

We therefore employed time-lapse microscopy to directly monitor co-cultures and evaluate whether cell competition may be taking place. For this assay, WT (depicted in green) and BaxKD (depicted in red) HFSCs were seeded in co-culture at a 1:1 ratio (Fig. 2e) and monitored daily until a high degree of cell-cell contact between populations was observed (5DPS), after which live imaging began. This revealed that within 15 h, nearly 60% of the WT population was eliminated within the immediate vicinity of BaxKD cells through a manner that appeared contact-dependent (Fig. 2f-h). Upon closer observation, these WT cells adjacent to BaxKD cells displayed apoptotic morphology including blebbing and detachment (Fig. 2f, g and Supplementary Movie 1; BaxKD depicted in red, WT depicted in blue). Furthermore, completion of apoptosis was found to occur as early as ~1 h and within ~3 h from observed contact, in contrast to ~7 h for WT cells surrounded by other WT counterparts (Supplementary Fig. 2c). These data suggest that BaxKD HFSCs can behave as super competitors, eliminating otherwise viable WT HFSCs.

We next examined whether apoptotic-resistant BaxKD cells drive the elimination of WT neighbors through non-autonomous induction of apoptosis. IF staining of co-cultures against cCp3 revealed the presence of apoptotic WT cells adjacent to BaxKD cells (Fig. 2i). However, to assess whether these cells were undergoing apoptosis through an extrinsic factor, we performed IF staining of co-cultures against cleaved Caspase-8 (cCp8) (Fig. 2j; Supplementary Fig. 2d). As cCp8 is predominantly activated under the extrinsic apoptotic cascade, it can potentially be used as an indicator of extrinsic apoptosis induction. This revealed that nearly all cCp8^+^ cells were WT and not BaxKD (Fig. 2k). Furthermore, positional analysis of WT cells with respect to BaxKD cells revealed that over 85% of cCp8^+^ WT cells were immediately adjacent to BaxKD cells, compared to only ~8% of cCp8^-^ WT cells (Fig. 2l), suggesting active cell elimination of WT cells may occur through apoptotic induction. Taken together, these findings indicate that Bax-depletion is sufficient to render HFSCs as super competitors that can eliminate their normal counterparts through induction of apoptosis.

While elimination of WT cells appeared to occur in a contact-dependent manner, we wanted to examine any potential role of factors secreted from BaxKD cells. To address this, WT HFSCs were treated daily with conditioned media (CM) harvested from BaxKD or WT HFSCs for 7 days. However, no apparent morphological changes indicative of cell death were observed (Supplementary Fig. 2e). To further investigate this, WT or BaxKD HFSCs were treated with CM harvested from competing WT+BaxKD HFSC co-cultures (CC) alongside WT or BaxKD media controls for up to 24 h^34^. However, IF of treated cells against cCp8 (Supplementary Fig. 2f) showed no significant changes (Supplementary Fig. 2g), suggesting that cell competition is not mediated via released factors.

### Winner cells harness TNFα for loser cell elimination

**Fig. 3:**
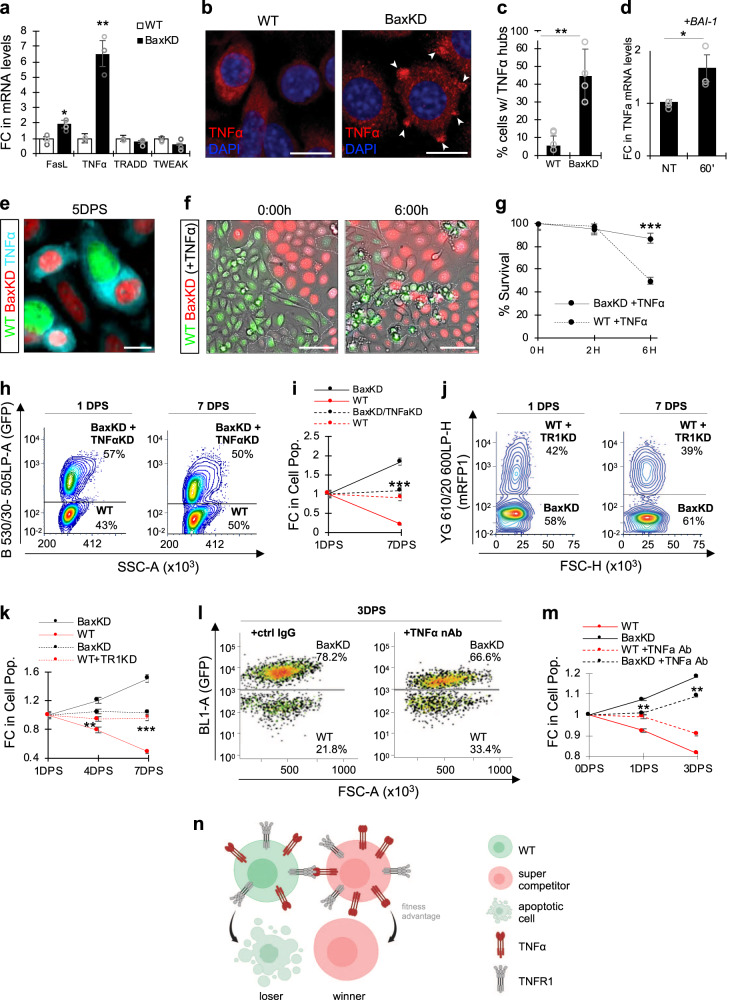
Winner cells harness TNFα for elimination of loser cells. **a** FC in mRNA levels between WT and BaxKD cells. **b** IF against TNFα in WT and BaxKD cells. Arrowheads denote membranal TNFα (mTNFα) hubs. **c** Percentage of cells with mTNFα hubs based on (**b**). More than 130 cells from 5 randomly selected fields across n = 3 biological replicates examined per condition. **d** FC in TNFα mRNA levels after 60 min of BAI-1 treatment. **e** IF against TNFα in WT and BaxKD co-cultures 5DPS. **f** Live BF and fluorescent imaging of WT and BaxKD co-cultures after 6 h of TNFα treatment (dashed lined indicates population boundaries). **g** Percentage of cell survival based on (**f**). 12 fields (as depicted in **f**) across 3 biological replicates examined. **h** FACS plots depicting WT and BaxKD+TNFαKD co-cultures 1 and 7DPS. **i** FC in cell populations based on (**h**). Asterisks represent comparison between the FC in BaxKD+TNFαKD co-cultured with WT HFSCs versus control BaxKD co-cultured with WT HFSCs. **j** FACS plots depicting WT+TR1KD (TNFα receptor 1 knockdown) and BaxKD co-cultures 1 and 7DPS. **k** FC in cell populations based on (**j**). Asterisks represent comparison between the FC in WT+TR1KD co-cultured with BaxKD HFSCs versus control WT co-cultured with BaxKD HFSCs. **l** FACS plots depicting WT and BaxKD co-cultures treated with the TNFα neutralizing antibody (nAb) Etanercept or control IgG after 1 and 3DPS. **m** FC in cell populations based on (**l**). Asterisks represent comparison between the FC in BaxKD HFSCs co-cultured with WT cells in TNFα nAb (Etanercept)-treated versus control IgG-treated WT and BaxKD co-cultures. **n** Schematic depicting super cell competition paradigm for WT (loser) and BaxKD (super competitor winner) cells. Here, a BaxKD cell of relatively greater fitness exhibits elevated levels of TNFα in the membrane compared to its WT neighbor, which ultimately facilitates the competitive elimination of the otherwise viable WT cell (created with BioRender.com). Scale bar represents 20 μm in **b**, **e**; 50 μm in **f**. *n* = 3 biological replicates per condition per experiment unless otherwise specified. Source data are provided as a Source Data file. Each experiment was repeated at least 2 times with similar results. All data are mean and ± SEM. P-values were determined by unpaired two-tailed t-test. *P* value < 0.05*, <0.01**, <0.001***.

We next aimed to identify the manner through which super competitor “winner” BaxKD HFSCs are able to eliminate neighboring “loser” WT HFSCs. Towards this, we examined the expression of various cell death ligands known to activate extrinsic apoptosis via quantitative real-time PCR (qPCR), which revealed a 6.5-fold increase in TNFα expression in BaxKD versus WT HFSCs (Fig. 3a). This was correlated with IF staining against TNFα, indicating higher expression in BaxKD HFSCs (Supplementary Fig. 3a). As our data suggested that cell competition was mediated via cell-cell contact, we checked whether TNFα could be detected at the cellular membrane (mTNFα), since it is more commonly cleaved from its transmembranal form and released into the cellular environment^35^. Interestingly, detailed examination of IF staining against TNFα revealed the localization of TNFα into compact “hubs” at the cellular membrane in BaxKD but not WT HFSCs (Fig. 3b, c). Likewise, IF staining against TNFα in live cells under non-permeabilizing conditions, which allows preferential membrane labeling, showed the presence of membrane TNFα in BaxKD cells (Supplementary Fig. 3b).

We next sought out to understand whether this change in TNFα occurs as an early event that directly follows Bax depletion, or as an indirect byproduct of upstream signaling or crosstalk that results in cellular state changes. To address this, we employed the Bax chemical channel blocker and inhibitor, BAI-1, which enabled us to assess the temporal dynamics of TNFα transcription and localization. After 30 min of treatment, TNFα transcription was elevated (Fig. 3d) and the presence of TNFα membranal hubs could be detected (Supplementary Fig. 3c), indicating this to be an early response to Bax depletion. Furthermore, these hubs could also be observed in BaxKD-WT co-cultures (Fig. 3e; Supplementary Fig. 3d).

We reasoned that if apoptotic induction of WT cells was executed via TNFα stimulation, additional stimulus with TNFα may exacerbate cell competition. We therefore treated co-cultures with TNFα and performed live imaging as previously described. Within 6 h, 50% of WT HFSCs were eliminated (Fig. 3f, g; Supplementary Movie 2; BaxKD depicted in red, WT depicted in blue), indicating that TNFα addition accelerated the elimination of WT cells. As a next step, we performed functional experiments to examine if BaxKD HFSCs indeed harness TNFα for the elimination of WT cells. We silenced TNFα (TNFαKD) (Table S1) in BaxKD HFSCs, which was estimated at ~70% (Supplementary Fig. 3e), followed by co-culturing with WT cells as previously described. Notably this relatively mild silencing was sufficient to reverse cell competition and resulted in no significant changes (Fig. 3h, i), unlike in control BaxKD and WT co-cultures (Supplementary Fig. 3f). As TNFα ligand binds to TNFα receptor 1 (TR1) to mediate signaling in a majority of cell types including HFSCs, we additionally silenced TR1 (Table S1) in WT cells (Supplementary Fig. 3g) followed by co-culturing with BaxKD cells. Providing further verification, FACS analysis revealed that TR1KD in WT cells was sufficient to prevent cell killing by BaxKD cells compared to control co-cultures (Fig. 3j, k; Supplementary Fig. 3h). We additionally performed experiments in which we treated co-cultures with the TNFα inhibitor Etanercept, which also showed significant attenuation of cell competition (Fig. 3l, m). Together, these data support the paradigm in which BaxKD cells induce WT cell elimination via membranal TNFα-TR1 signaling (Fig. 3n).

### Bax depletion results in p65 activation and TNFα elevation

As TNFα elevation was observed to be a relatively early event following Bax inhibition, we next wanted to investigate the mechanism through which this may occur. Although Bax activity and MOMP have predominantly been associated with apoptotic triggers, previous work has established the occurrence of minority MOMP^36^, in which mitochondria continuously release sublethal levels of their contents under steady-state conditions. We therefore reasoned that perhaps inhibition of Bax results in TNFα elevation through diminished release of some factor from the mitochondria.

**Fig. 4:**
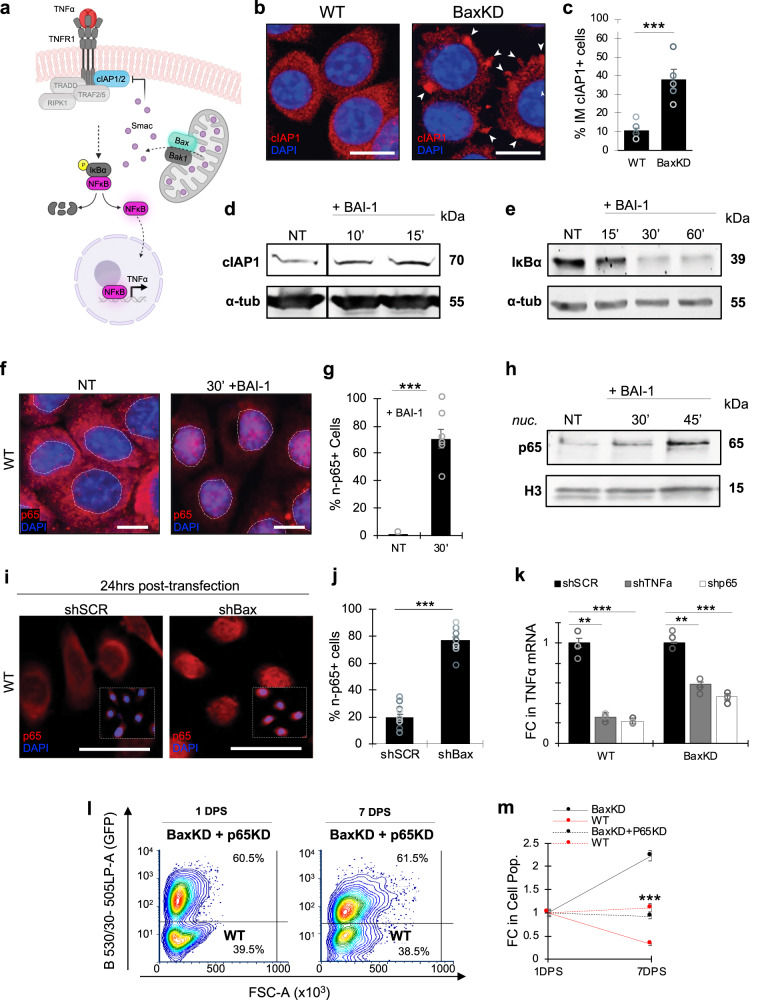
Bax inhibition results in activation of p65. **a** Schematic of proposed mechanism depicting the relationship between Bax inhibition, p65 activation, and TNFα elevation. In this model, under the presence of Bax, appropriate stimuli can enable the release of Smac from the mitochondria, thereby inhibiting cIAP1/2 activation and downstream NFκB activation and target gene expression (including TNFα). In the absence of Bax, cIAP1/2 may become activated and promote downstream NFκB activation and target gene expression (created with BioRender.com). **b** IF staining against cIAP1 in WT and BaxKD cells. Arrowheads denote cIAP1 inter-membranal (IM) hubs. **c** Percentage of IM cIAP1^+^ cells based on (**b**). 5 randomly selected fields of at least 350 cells and *n* = 3 biological replicates were examined per condition. **d** IB of WT HFSCs against cIAP1 and α-tubulin 10 and 15 min after BAI-1 treatment. **e** IB of WT HFSCs against IκBα and α-tubulin 15, 30, and 60 min after BAI-1 treatment. **f** IF staining against p65 in HFSCs 30 min after BAI-1 treatment. Dotted lines mark nuclear boundaries. **g** Percentage of nuclear (n)-p65^+^ cells based on (**f**). More than 450 cells across 6 randomly selected fields and *n* = 3 biological replicates were examined per condition. **h** IB against p65 and histone 3 (H3) in the nuclear fractions of WT HFSCs NT, 30, 45, and 60 min after BAI-1 treatment. **i** IF staining against p65 in WT HFSCs 24 h post-transfection with shSCR (left) and shBax (right). **j** Quantification of % of cells with nuclear p65 translocation based on (**i**). More than 250 cells across 8 randomly selected fields and *n* = 3 biological replicates evaluated per condition. **k** FC in mRNA levels in WT or BaxKD HFSCs transfected with shSCR, shTNFα, or shp65. **l** FACS plot depicting BaxKD+p65KD and WT co-cultures 1 and 7DPS. **m** FC in cell populations based on (**l**), representing significant reversal in cell competition. Asterisks represent comparison between the FC in WT and BaxKD+p65αKD co-cultures versus control WT and BaxKD co-cultures. Scale bar represents 20 μm in **b**, **f**; 50 μm in **i**. *n* = 3 biological replicates per condition per experiment unless otherwise specified. Source data are provided as a Source Data file. Each experiment was repeated at least 2 times with similar results. All data are mean and ± SEM. P-values were determined by unpaired two-tailed t-test. *P* value < 0.05*, <0.01**, <0.001***.

It has been previously shown that the inhibitor of apoptosis (IAP) antagonist Smac, which is released from the mitochondria in a Bax-dependent manner^37,38^, binds and inhibits cIAP1/2^39^. This can in turn prevent cIAP1/2 from translocating to the intracellular domain of TR1 and subsequently activating NF*κ*B signaling under appropriate conditions^40,41^. As TNFα is an established target gene of NF*κ*B, we hypothesized that diminished release of Smac may increase the availability of cIAP1/2 to induce NF*κ*B/p65 activation and downstream TNFα expression upon Bax inhibition (Fig. 4a). In support, previous work has also demonstrated that Smac deletion results in elevation of cIAP1/2 and p65 activation in tumorigenic cells^42^.

To examine this model, we first performed IF staining against Smac together with mitochondrial labeling (MitoTracker), which revealed a decrease in Smac localized outside of the mitochondria in BaxKD versus WT HFSCs (Supplementary Fig. 4a). As Smac inhibits cIAPs by binding and preventing translocation, we next assessed whether diminished cytosolic Smac in BaxKD cells would correlate with changes in cIAP localization. In support of this, we found a significant increase in cells with localization of cIAP1 in the membranal vicinity in BaxKD HFSCs (Fig. 4b, c; Supplementary Fig. 4b). Furthermore, immunoblotting revealed increased cIAP1 levels from 10 min after BAI-1 treatment (Fig. 4d; Supplementary Fig. 4c).

The binding of cIAP to TR1 triggers activation of a cascade, ultimately leading to the phosphorylation and degradation of the NF*κ*B inhibitor I*κ*Bα^43^. To further analyze whether the temporal dynamics of these changes were consistent with being the direct result of Bax inhibition, cells were treated with BAI-1 and assayed for changes over time. Immunoblotting against I*κ*Bα revealed a decrease in the levels within 15 min of treatment, followed by a further decline (Fig. 4e).

As the translocation of RelA/p65 into the nucleus serves as an indication of NF*κ*B activation, we performed IF staining against p65 after BAI-1 treatment and found nuclear localization (n-p65) in ~62% of cells in contrast to 0% in untreated controls within 30 min (Fig. 4f, g; Supplementary Fig. 4d). These results were verified via nuclear fractionation followed by immunoblotting (Fig. 4h; Supplementary Fig. 4e) and are temporally consistent with changes in upstream protein levels. However, BaxKD cells displayed no significant changes in n-p65 compared to controls upon treatment with BAI-1, verifying the treatment specificity (Supplementary Fig. 4f, g). To compare the extent of n-p65 between Bax chemical inhibition versus mRNA silencing, we performed transient knockdown against Bax (Table S1) in WT HFSCs and evaluated them 24-hours post-transfection, by which point a significant extent of silencing is expected to occur. In agreement with our earlier findings, ~77% of cells versus ~19.4% in shSCR controls were n-p65^+^ (Fig. 4i, j). We therefore evaluated whether similar trends can be observed upon targeting of Smac (Table S1) in the same manner, which revealed n-p65 in ~64% of WT HFSCs post-transfection (Supplementary Fig. 4h, i). Notably, upon treatment of stably silenced TR1 HFSCs (TR1KD) (Table S1) with BAI-1, we found no significant difference in the levels of cIAP and I*κ*Bα (Supplementary Fig. 4j, k), as well as nuclear p65 translocation (Supplementary Fig. 4l, m), supporting the role of TR1 within this model.

To next determine whether TNFα expression is dependent upon NF*κ*B signaling, we performed transfections that enabled transient knockdown of TNFα and p65 alongside SCR controls (Table S1) in individual WT and BaxKD cultures and subsequently assessed TNFα mRNA levels. Our data revealed that the knockdown of RelA/p65 reduced TNFα mRNA comparably to knockdown of TNFα (Fig. 4k), indicating dependency of TNFα expression on p65. Finally, as p65 was found to regulate expression of TNFα, we next wanted to examine its requirement for cell competition to occur. Towards this, we silenced the NF*κ*B component RelA/p65 (p65KD) in BaxKD cells (Supplementary Fig. 4n) and co-cultured these with WT cells. FACS analysis revealed significant reversal in cell competition (Fig. 4l, m), indicating its requirement for cell competition to occur. Collectively, these data show that Bax depletion directly leads to the activation of p65, which is necessary for TNFα mediated cell competition.

As we have observed potent p65 activation upon inhibition of upstream pro-apoptotic factors, we wondered whether positive regulation of the pro-survival factor, Bcl2, may similarly activate p65. To investigate this, WT HFSCs were transfected as previously described with a Bcl2 overexpression (Bcl2OE) vector (Table S1), which resulted in translocation of p65 in ~62% of cells (Supplementary Fig. 4o, p). Next, we examined whether overexpression of an anti-apoptotic factor rather than silencing of a pro-apoptotic factor would have any functional consequences comparable to Bax inhibition. Towards this, we performed colony formation assays of HFSCs overexpressing Bcl2 (Supplementary Fig. 5a), which displayed a ~ 1.3-fold, 4-fold, and ~5-fold increase in number of colonies per field, colony area, and cell number, respectively (Supplementary Fig. 5b-f), as well as a ~ 1.4-fold increase in the number of Ki-67^+^ cells (Supplementary Fig. 5g, h). Upon examining whether this was correlated with changes in TNFα expression, we found only a slight elevation of TNFα in Bcl2OE HFSCs (Supplementary Fig. 5i). Although these changes were not strong, we examined whether silencing of TNFα in Bcl2OE cells would affect colony formation and found an over 2-fold decrease in the number of colonies formed (Supplementary Fig. 5i-k). However, no significant changes in the colony size or cellular density within the colonies was observed (Supplementary Fig. 5l-n), indicating that while TNFα may play a role in colony establishment, it is unlikely to be responsible in the expansion of these cells. We next seeded co-cultures to investigate whether Bcl2OE cells exhibit a competitive advantage over WT counterparts. However, FACS analysis did not show striking enrichment of Bcl2OE cells (~1.1-fold) 7DPS (Supplementary Fig. 5o, p), unlike in the case of BaxKD cells. It may be valuable for future work to investigate the possibilities of whether this could be due to the relatively modest changes in Bcl2 levels upon ectopic expression and TNFα elevation, or independent roles of Bax and Bcl2 as well as differential effects of targeting pro-apoptotic versus anti-apoptotic factors.

### Winner cells can differentially respond to TNFα via TNFR2

**Fig. 5:**
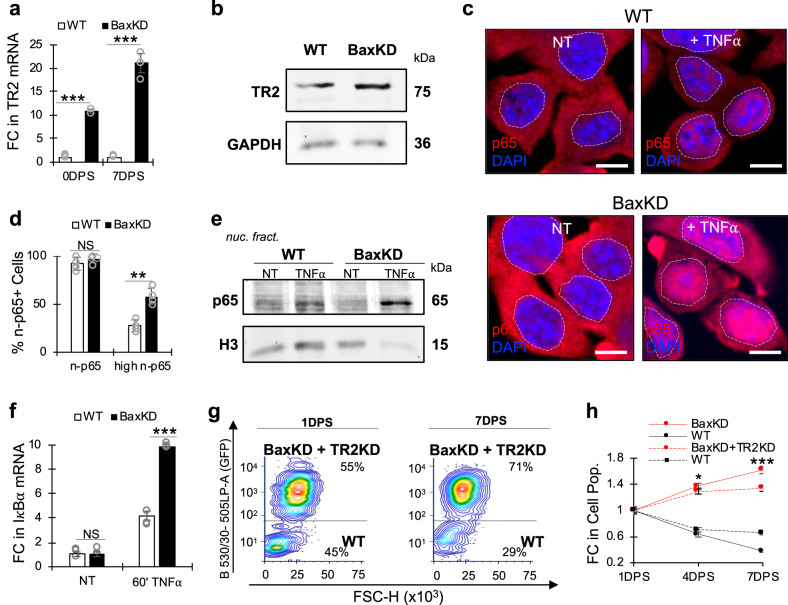
Bax-depleted cells differentially respond to TNFα stimulation. **a** FC in mRNA of Tnfr2 (TR2) in WT and BaxKD cells in co-culture at 0 and 7DPS. **b** IB against TR2 and GAPDH in WT and BaxKD HFSCs. **c** IF staining against p65 in WT (top) and BaxKD (bottom) HFSCs 5 min after TNFα treatment. Dotted lines mark nuclear boundaries. **d** Percentage of n-p65^+^ cells and high expressing n-p65^+^ cells based on (**c**). More than 170 cells across 4 randomly selected fields from *n* = 4 biological replicates (1 field per replicate) were examined for each condition. **e** IB against p65 and histone 3 (H3) in the nuclear fraction of WT HFSCs or BaxKD HFSCs 5 min after TNFα treatment. **f** FC in mRNA levels of IκBα in WT or BaxKD cells 1 h after TNFα treatment. **g** FACS plot depicting BaxKD+TR2KD and WT co-cultures 1 and 7DPS. **h** FC in cell populations based on (**g**). Asterisks represent comparison between the FC in WT and BaxKD+TR2KD (TNFα receptor 2 knockdown) co-cultures versus control WT and BaxKD co-cultures. Scale bar represents 15 μm in **c**. *n* = 3 biological replicates per condition per experiment unless otherwise specified. Source data are provided as a Source Data file. Each experiment was repeated at least 2 times with similar results. All data are mean and ± SEM. P-values were determined by unpaired two-tailed t-test. *P* value < 0.05*, <0.01**, <0.001***.

While the cytokine TNFα can serve as a death ligand and activate extrinsic apoptosis under certain conditions, it can also function to activate NF*κ*B pro-survival signaling^44^. Its ability to trigger diverse cellular responses, such as pro-survival and proliferative signaling, as well as inflammatory and apoptotic signaling is dependent upon various factors. These include TNFα dosage, duration and cellular status prior to exposure, whether it is in membranal or solubilized form, and critically, the ratios of TNFα receptor 1 or 2 (TR1 or TR2)^45–49^. While TR1 is ubiquitously expressed across most cell types, TR2 is primarily found in immune cells, endothelial cells, neural cells, mesenchymal SCs, and some types of tumor cells^50^. Although both receptor subtypes are important for inflammatory signaling, TR1 can directly trigger apoptotic signaling, while TR2 lacks an intracellular death domain and also triggers potent anti-inflammatory and pro-survival signaling^51–54^. We therefore analyzed SC RNA-seq data of adult mouse epidermis to compare the expression of TNFα receptor subtypes in CD34^+^ HFSCs, which revealed significant expression of TR1 compared to TR2 (Supplementary Fig. 6a). Interestingly, qPCR for TR1 and TR2 mRNA levels in WT and BaxKD co-cultures revealed over 10 and 20-fold increase in TR2 but not TR1 expression in BaxKD cells at 0 and 7DPS respectively (Fig. 5a; Supplementary Fig. 6b). These changes in basal TR2 mRNA levels were further supported by immunoblotting against TR2 (Fig. 5b; Supplementary Fig. 6c). To determine whether this correlates with distinct cellular responses to TNFα stimulation, we quantified the degree of NF*κ*B activation in BaxKD versus WT cells. IF staining against p65 revealed a greater percentage of BaxKD exhibiting high nuclear p65 localization compared to WT cells (Fig. 5c, d; Supplementary Fig. 6d). Similarly, nuclear fractionation followed by immunoblotting against p65 revealed a greater degree of RelA/p65 translocation in BaxKD cells upon TNFα stimulation (Fig. 5e; Supplementary Fig. 6e).

We further investigated the mRNA expression of the NF*κ*B transcriptional target I*κ*Bα after stimulation with TNFα and found that while both WT and BaxKD exhibited elevation in I*κ*Bα expression, BaxKD cells exhibited a significantly stronger upregulation in I*κ*Bα (Fig. 5f). Furthermore, as BaxKD cells acquire ectopic expression of TR2, we speculate that this may enable them to not only survive contact from neighboring BaxKD cells harboring mTNFα, but additionally fuel them to further propagate. Previous studies have also demonstrated a significantly greater binding affinity of TR2 with membranal TNFα (mTNFα) compared to solubilized TNFα, which supports this notion^50,55–57^. We therefore performed knockdown of TR2 (Table S1) in BaxKD cells, which resulted in 70% silencing (Supplementary Fig. 6f). FACS analysis of co-cultures revealed that the mild silencing of TR2 had a significant impairment in the ability of BaxKD cells to continue propagating once cells reached densities that enabled a greater degree of contact (Fig. 5g, h; Supplementary Fig. 6g). We also tested how the depletion of TR2 in BaxKD cells would affect responses to TNFα stimulation. Nuclear fractionation followed by immunoblotting against p65 revealed that while p65 translocated into the nucleus in control BaxKD cells upon stimulation, this response was not observed in TR2 silenced cells (Supplementary Fig. 6h, i).

### Apoptotic dysregulation alters tissue homeostasis in vivo

**Fig. 6:**
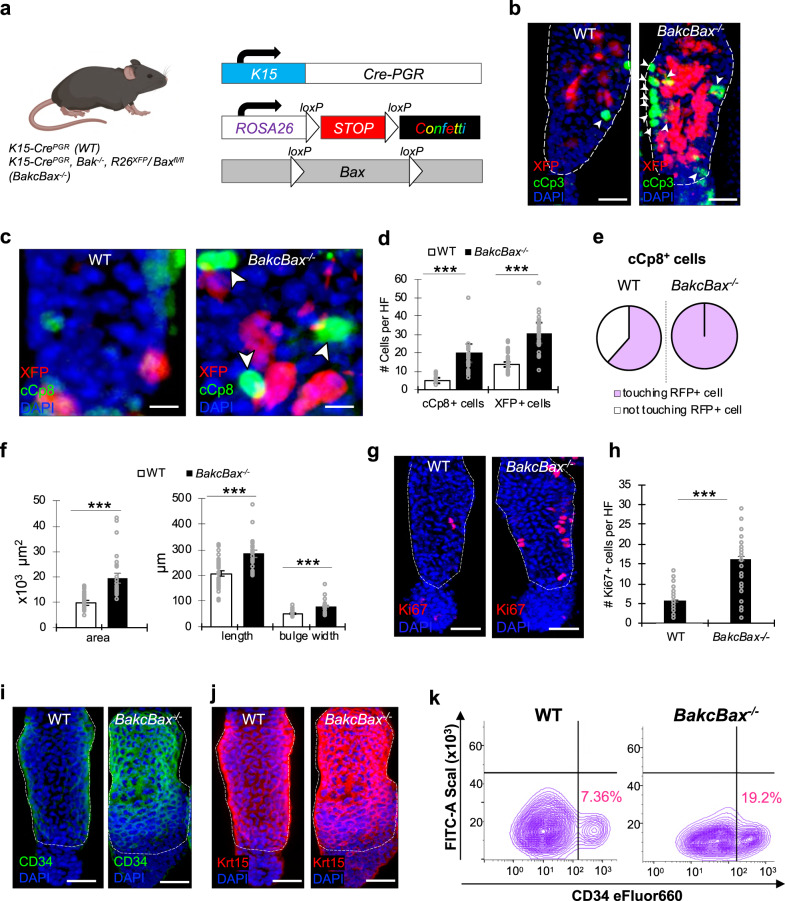
Bax-depleted HFs exhibit increased non-cell autonomous apoptotic induction, HFSC niche expansion, and hypertrophic morphology in vivo. **a** Schematic depicting conditional knockout mouse model on *BakKO* background used in experiments (*BakcBax*^−/−^) (created with BioRender.com). **b**, **c** IF staining against cCp3 (**b**) and cCp8 (**c**) in WT or *BakcBax*^−/−^ HFs with fluorescent labeling of induced cells (depicted as XFP; red). Arrowheads denote apoptotic cells adjacent to XFP^+^ cells. Dotted lines mark HFSC bulge region. **d** Quantification of the number of cCp8^+^ or XFP^+^ cells per HF in WT or *BakcBax*^−/−^ mice based on (**c**). *n* = 3 biological replicates for both WT and *BakcBax*^−/−^ with a minimum of 25 HFs examined per condition. **e** Percentage of cCp8^+^ cells in either WT or *BakcBax*^−/−^ HFs in proximity with an induced (XFP) cell based on (**c**). **f** HF size metrics including area, length, and bulge width in WT and *BakcBax*^−/−^ HFs. *n* = 3 biological replicates for both WT and *BakcBax*^−/−^ with a minimum of 25 HFs examined per condition. **g** IF staining against Ki-67 in HFs from WT and *BakcBax*^−/−^ TWMs. Dotted lines mark the HFSC bulge region. **h** Quantification of number of Ki-67^+^ cells per HF in WT and *BakcBax*^−/−^ TWMs based on (**g**). *n* = 3 biological samples for WT and *BakcBax*^−/−^ with a minimum of 30 HFs examined per condition. **i**, **j** IF staining against CD34 (**i**) or Keratin15 (Krt15) (**j**) in WT and *BakcBax*^−/−^ HFs. Dashed lines denote the HFSC niche. **k** FACS analysis quantifying the CD34^+^ HFSC population derived from Integrin-α6^+^/ScaI^-^ parent epidermal pool in WT versus *BakcBax*^−/−^ mice (*n* = 3 pooled mice). Scale bar represents 25 μm in **b, g, i, j**; 10 μm in **c**. Each experiment was repeated at least 2 times with similar results. *n* = 3 biological replicates per condition per experiment unless otherwise specified. Source data are provided as a Source Data file. All data are mean and ± SEM. P-values were determined by unpaired two-tailed t-test. *P* value < 0.05*, <0.01**, <0.001***.

As a next step, we investigated whether apoptotic dysregulation functionally affects tissue homeostasis in vivo. Although it has previously been established that Bax and Bak exhibit some degree of overlapping and compensatory roles, *Bak*^−/−^ mice appear phenotypically normal while *Bax*^−/−^ mice exhibit abnormalities including male sterility, mild lymphoid hyperplasia, and an increased number of neurons^22,58,59^. We therefore utilized mice harboring *Bak* knockout together with K15 promoter-driven CRE-PGR deletion of Bax (*BakcBax*^−/−^) and confetti (XFP) expression (Fig. 6a; Supplementary Fig. 7a), enabling examination of the role of Bax in HFSCs while preventing compensation by Bak. Conditional deletion of Bax was induced in 8-week-old mice, when in the telogen phase, through topical and subcutaneous administration of RU486 for 5 days followed by a 5-day chase period, after which tail skin of mice was harvested for further analysis (Supplementary Fig. 7b, c). IF staining against p65 and TNFα corroborated our previous findings, indicating increased levels in *Bax*-depleted HFs (Supplementary Fig. 7d, e). To next evaluate whether any indicators of similar cell competition-like phenomenon may occur in vivo within *Bax*-depleted HFs, we performed IF staining against cCp3. In complete agreement with our in vitro studies, cCp3^+^ cells were present in the immediate vicinity of *Bax*-depleted cells (Fig. 6b) and TUNEL staining confirmed these cells to be apoptotic (Supplementary Fig. 7f). Additionally, IF staining against cCp8 also indicated that ~26% of fluorescently labeled cells in WT HFs versus ~7% in *BakcBax*^−/−^ HFs were cCp8^+^ (Fig. 6c; Supplementary Fig. 6g, h). Assessment of the number of cCp8^+^ or XFP^+^ cells per HF revealed a greater number of both cCp8^+^ and XFP^+^ cells in *Bax*-depleted HFs (Fig. 6d), suggesting a correlation between the increase of XFP^+^ and cCp8^+^ cells. Furthermore, positional analysis of cCp8^+^ cells revealed that 100% of cCp8^+^ cells in *BakcBax*^−/−^ HFs were adjacent to a labeled (*Bax*-depleted) cell, compared with 62% in WT HFs (Fig. 6e). Together, these data may suggest the presence of non-autonomous apoptotic induction similar to what we previously described in our competitive cell assays.

Upon morphological observation, *BakcBax*^−/−^ HFs exhibited larger overall HF size compared to HFs of WT as well as *Bak*^*-/-*^ mice (Fig. 6f; Supplementary Fig. 7i). Consistent with our in vitro results, *Bax*-depleted HFs also displayed an increase in the number of Ki-67^+^ proliferating cells (Fig. 6g, h), as well as higher expression of CD34^+^ and Keratin (Krt15)^+^ HFSC markers (Fig. 6i, j). This was verified via FACS analysis, which revealed a 2.6-fold increase in the CD34^+^ HFSC population in *BakcBax*^−/−^ mice (Fig. 6k). Moreover, in many cases, *Bax*-depleted HFs additionally displayed tissue hypertrophy (Fig. 6i, j; Supplementary Fig. 7i), implicating an important homeostatic role for Bax in the maintenance of the HFSC niche.

Previously, it has been shown that a higher degree of proliferating cells can be characteristic of HF induction into anagen^60^. To examine whether inhibition of Bax may affect hair cycle dynamics, telogenic mice treated with BAI-1 were waxed in order to induce entry into anagen^61^ (Supplementary Fig. 8a). Daily monitoring of coat color until 9 days post-depilation revealed similar patterns of skin darkening between control and BAI-1 treated mice (Supplementary Fig. 8b), and histological analysis of skin sections 9 days post-depilation similarly showed no significant effect on the morphology and length of HFs (Supplementary Fig. 8c, d), suggesting that it does not affect the HF cycle.

### Bax depletion facilitates tissue repair and regeneration

**Fig. 7:**
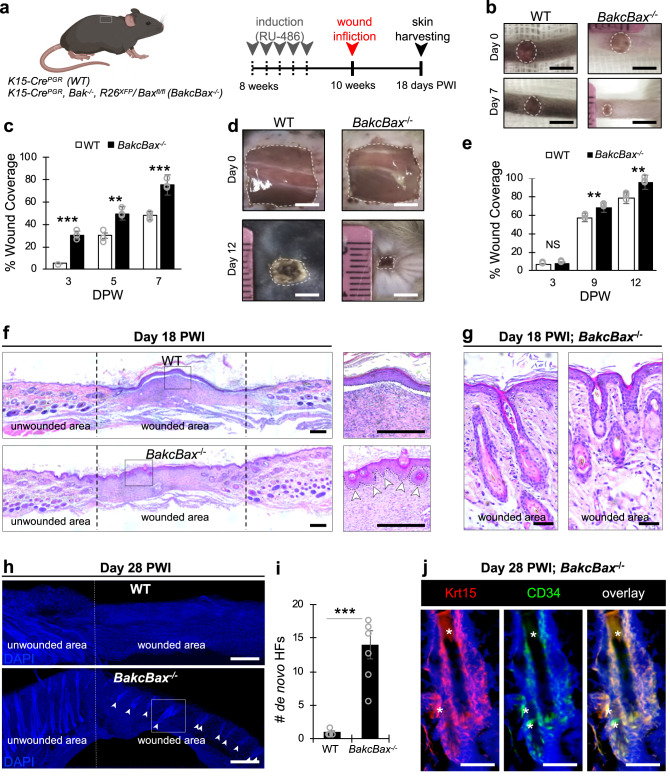
Bax depletion results in accelerated wound healing and formation of de novo hair follicles in vivo. **a** Schematic of wounding experiments. PWI= post wound induction. Dashed shapes denote tail and dorsal wound infliction sites (created with BioRender.com). **b** Images of tail epidermal wounds in WT and *BakcBax*^−/−^ mice 0 and 7 days PWI. **c** Percentage of wound coverage based on (**b**). *n* = 3 biological replicates for both WT and *BakcBax*^−/−^ mice. **d** Images of dorsal epidermal wounds in WT and *BakcBax*^−/−^ mice 0 and 12 days PWI. **e** Percentage of wound coverage based on (**d**). *n* = 3 biological replicates for both WT and *BakcBax*^−/−^ mice. **f** Hematoxylin and Eosin (H&E) staining of WT and *BakcBax*^−/−^ dorsal skin sections from within the wound bed 18 days PWI. Panels to the right represent boxed regions, dashed lines denote wound borders, and arrowheads denote formation of de novo HFs. **g** H&E staining of *BakcBax*^−/−^ dorsal skin sections from within the wound bed 18 days PWI. **h** DAPI staining of WT and *BakcBax*^−/−^ dorsal skin sections from within the wound bed 28 days PWI. Dashed lines denote wounded region and arrowheads denote de novo HFs. **i** Quantification of number of de novo HFs in WT and *BakcBax*^−/−^ mice based on (**h**). *n* = 3 biological replicates for both WT and *BakcBax*^−/−^ mice, with the total number of HFs evaluated across 3 regions (as represented in the image). **j** IF staining against Krt15 and CD34 depicting boxed region in (**h**). Asterisks denote autofluorescence. Scale bar represents 4 mm in **b, d**; 100 μm in **f**; 50 μm in **g, j**; 150 μm in **h**. Each experiment was repeated at least 2 times with similar results. *n* = 3 biological replicates per condition per experiment unless otherwise specified. Source data are provided as a Source Data file. All data are mean and ± SEM. P-values were determined by unpaired two-tailed t-test. *P* value < 0.05*, <0.01**, <0.001***.

While HFSCs do not contribute to the epidermis during homeostasis, they play a critical role in facilitating repair and regeneration following epidermal wound infliction^62–65^. Therefore, to investigate the functional consequences of apoptotic dysregulation in these SCs, we performed full-thickness epidermal skin excisions in the tail and dorsal skin (Fig. 7a). Monitoring of wound closure revealed significantly accelerated healing dynamics in *BakcBax*^−/−^ mice, with 75% tail wound closure versus 48% in WT mice 7 days post wound infliction (PWI) (Fig. 7b, c), and 96% dorsal wound closure compared to 79% in WT mice 12 days PWI (Fig. 7d, e). Since a critical hallmark of SCs is the ability to facilitate tissue replenishment and regeneration post-injury, we next evaluated whether Bax depletion was consequential to this process. Typically, full-excision epidermal wounds result in scar formation, however in rare cases, minimal formation of HFs or similar de novo structures have been reported^63,66,67^. Analysis of histological hematoxylin and eosin (H&E) staining from within wound beds of dorsal skin 18 days PWI revealed epidermal invaginations reminiscent of HF placodes together with HFs in *BakcBax*^−/−^ but not control mice^63,67^ (Fig. 7f, g; Supplementary Fig. 9a, b). By 28 days PWI, a 14-fold increase in fully formed HF-like structures could be observed from the wounded region in *BakcBax*^−/−^ mice (Fig. 7h, i). Furthermore, IF staining against HFSC markers CD34 and Krt15 18 and 28 days PWI further indicated these regenerated HF structures indeed encompass HFSCs (Fig. 7j; Supplementary Fig. 9c, d). This is in agreement with previous works that implicated deletion of pro-apoptotic ARTS or Casapse-9 in promoting wound healing and HF regeneration^9,66^.

Evaluating unwounded dorsal skin regions, we observed Bax expression in HFs of WT and, expectedly to a lesser extent, *BakcBax*^−/−^ mice (Supplementary Fig. 9e). However, while some *Bax* expression was observed in HFs of *BakcBax*^−/−^ mice, likely due to partial recombination, Bax expression was completely absent from the majority of regenerated HFs, with only ~14% of HFs expressing Bax compared to 89% in HFs from unwounded in *BakcBax*^−/−^ skin regions (Supplementary Fig. 9f, g). These results suggest that the enhanced expansion and relatively increased fitness potential of Bax depleted HFSCs may facilitate wound repair and SC-dependent HF regeneration in response to injury.

**Fig. 8:**
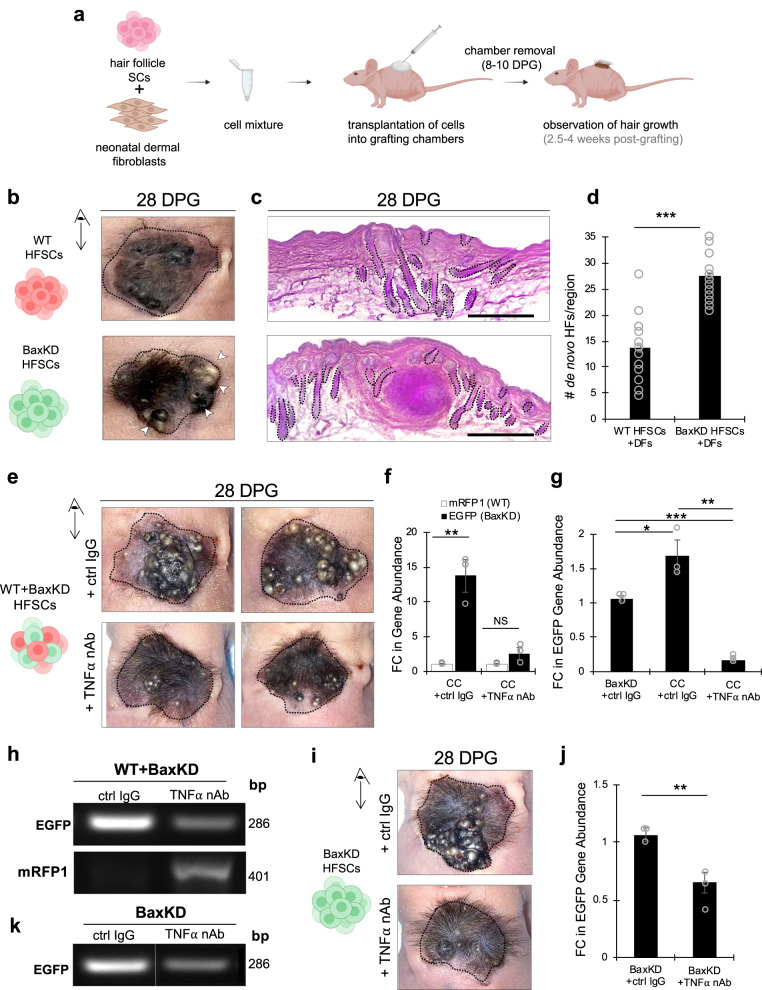
Transplanted BaxKD HFSCs exhibit enhanced HF generation capacity and outcompete WT HFSCs in a manner attenuated by TNFα neutralization in vivo. **a** Schematic depicting the grafting of HFSCs and neonatal dermal fibroblasts (DFs) into nude mice. **b** Images representing hair growth in grafts derived from WT (top) or BaxKD (bottom) HFSCs 28 days post-grafting (DPG). **c** H&E staining of graft sections derived from WT (top) or BaxKD (bottom) HFSCs 28 DPG. **d** Quantification of number of de novo HFs per region based on (**c**). **e** Images representing hair growth in grafts derived from mixed WT+BaxKD HFSCs treated with control IgG (top) or TNFα neutralizing antibody (TNFα nAb) (bottom) 28 days post-grafting (DPG). **f** FC of EGFP versus mRFP1 genes from qPCR of genomic DNA (gDNA) of WT+BaxKD HFSC-derived grafts (cell competition "CC" condition) treated with control IgG or TNFα nAb (EGFP and mRFP1 directly correspond to BaxKD and WT HFSCs respectively). **g** FC of EGFP gene from qPCR of genomic DNA (gDNA) derived from BaxKD +ctrl IgG, WT+BaxKD +ctrl IgG, or WT+BaxKD + TNFα nAb HFSC-derived grafts. **h** Nucleic acid gel depicting EGFP (top) and mRFP1(bottom) amplicons from gDNA of grafts treated with control IgG or TNFα nAb. **i** Images representing hair growth in grafts derived from BaxKD HFSCs treated with control IgG (top) or TNFα nAb (bottom) 28 days post-grafting (DPG). **j** FC of EGFP gene from qPCR of genomic DNA (gDNA) of BaxKD HFSC-derived grafts treated with control IgG or TNFα nAb. **k** Nucleic acid gel depicting EGFP amplicons from gDNA of BaxKD HFSC-derived grafts treated with control IgG or TNFα nAb. Schematics in panels **a**, **b**, **e**, **i** were created with BioRender.com. Scale bar represents 500 μm for **c**. *n* = 3 biological replicates per condition per experiment unless otherwise specified. Source data are provided as a Source Data file. Each experiment was repeated at least 2 times with similar results. All data are mean and ± SEM. *P* values were determined by unpaired two-tailed t-test. *P* value < 0.05*, <0.01**, <0.001***.

Finally, we sought to directly evaluate the contribution of BaxKD versus WT HFSCs to hair and skin regeneration by performing in vivo transplantation assays. This assay, which has typically been performed by combining extracted whole epidermal cell fractions and neonatal dermal fibroblasts followed by grafting onto nude mice^68,69^, provides an excellent and robust model for examining the functional consequences of genetic manipulation on epidermal or dermal population behaviors. However, given their multipotency and ability to contribute to regenerative processes, we reasoned that HFSCs alone might be utilized in place of whole epidermal cell fractions^70^. This could enable the direct evaluation and comparison of HFSC populations in hair formation without the support of other epidermal cell populations. Harnessing this approach (Fig. 8a), grafting of cultured HFSCs and freshly isolated neonatal dermal fibroblasts resulted in the robust generation of hairs within 2.5–4 weeks post-grafting, unlike in controls of DFs alone (Fig. 8b; Supplementary Fig. 10a, b). Notably, grafts derived from BaxKD and not WT HFSCs exhibited early hair growths from 18 DPG, with a significant difference in the number of hair follicles observed visibly and in H&E graft sections after 28 DPG as well as after 56 DPG (Fig. 8c, d; Supplementary Fig. 10a-c). In addition, BaxKD HFSC-derived grafts also developed abnormal hyperplastic growths in parallel with a high degree of hair formation in a majority of grafts (Fig. 8b, bottom panel, denoted by arrowheads). H&E staining of these abnormal structures within grafts revealed morphologies reminiscent of keratin horns or pearls observed in pathologies such as squamous cell carcinoma (SCC) (Supplementary Fig. 10d top panel). We speculate that this phenotype may perhaps be representative of hyperproliferation or improper elimination of BaxKD HFSCs during hair formation. Nevertheless, we believe these results further indicate the potential for BaxKD cells in driving aberrant phenotypes and represent a valuable avenue for future investigation.

Furthermore, to investigate whether BaxKD HFSCs can outcompete WT HFSCs in vivo, we performed competitive grafting experiments in which we transplanted each cell population at a 1:1 ratio. Phenotypically, mixed population grafts strongly resembled BaxKD HFSC derived grafts in their extent of hair growth as well as the formation of abnormal hyperplastic structures (Fig. 8e, top panel; Supplementary Fig. 10d, bottom panel), consistent with the prevalence of BaxKD cells under these conditions. To directly quantify the relative contribution of each cell population, we performed qPCR analysis of EGFP and mRFP1 genes, corresponding to BaxKD and WT HFSCs, respectively, in genomic DNA (gDNA) of graft samples harvested 28 DPG. This revealed an over 15-fold increase in EGFP (Fig. 8f), indicating a competitive phenotype. Notably, the degree of EGFP was greater in mixed population versus BaxKD-only grafts, suggesting that the presence of WT HFSCs may promote the expansion of BaxKD HFSCs (Fig. 8g; Supplementary Fig. 10e).

To next assess whether TNFα inhibition could reverse competition, grafts were treated with TNFα neutralizing antibody (nAb) or an IgG control post-transplantation for 5 consecutive days (days 0 to 4 post-grafting) and monitored over time (Fig. 8e). Analysis revealed a 2.7-fold difference in EGFP relative to mRFP1 (Fig. 8f) and marked reduction in EGFP abundance relative to control IgG samples (Fig. 8g), alongside an increase in mRFP1 compared to IgG controls (Fig. 8h). Additional treatment of BaxKD-only derived grafts with TNFα nAb also revealed a decrease in the extent of BaxKD HFSC contribution compared to IgG controls, (Fig. 8i-k). Interestingly, TNFα nAb-treated samples in both mixed population and BaxKD-only HFSC derived grafts appeared to have a decrease in the overall surface area of abnormal hyperplastic growths (Fig. 8e, i, bottom panels). These observations are in line with our previous results and further point to an important role for TNFα in the propagation of BaxKD HFSCs and promotion of WT cell elimination.

Collectively, our findings indicate that Bax plays a critical role in regulating HFSC homeostasis and cell elimination both autonomously and non-autonomously. Furthermore, depletion of Bax leads to apoptotic resistance, enhanced proliferation, and activation of p65, which collectively promote Bax-depleted cells to behave as super competitive “winners.” Harnessing TNFα, Bax-depleted HFSCs are capable of eliminating healthy neighboring cells in a contact-dependent manner, while evading elimination themselves. Functionally, these changes lead to HFSC expansion both in vitro and in vivo, catalyzing enhanced wound repair, tissue regeneration, and aberrant tissue morphologies.

## Discussion

Since SCs harness apoptosis for normal development as well as homeostasis of various adult tissues, it is critical to unveil the consequences of apoptotic dysregulation on the cellular and tissue scale. Here, we show that in conjunction to promoting apoptotic resistance, Bax depletion ultimately causes alterations in cellular state, rendering cells as super competitors capable of eliminating otherwise viable WT neighbors. Previously, many studies have identified genotypes that result in imbalances in cellular fitness and contribute to cell competition, as well as mechanisms that enable winner cells to execute cell competition^31–33,71–75^. Furthermore, this phenomenon has been found to play a fundamental role during development and homeostasis of various tissues including the skin^76,77^, as well as during both disease prevention and onset. However, the cell-cell interactions responsible for loser cell elimination need to be elucidated more extensively in mammalian contexts. We present a mechanism of cell competition that relies on TNFα-TNFR1 interactions between winner and loser SCs, and possibly the progeny of these SCs, to facilitate elimination, finding that winner cells rely on p65-dependent TNFα expression to execute cell elimination. Furthermore, we propose that upon depletion of Bax and impairment in the release of IAP antagonists from the mitochondria, IAPs are available to drive downstream activation of p65 and expression of the NF*κ*B target gene TNFα. While loser cells undergo TNFα-dependent elimination, winner cells are able to survive under the same conditions and activate NF*κ*B signaling to a greater extent. We speculate that this may partially be due to the differential expression of TNFR2 in winner cells, which engages TNFα to preferentially induce pro-survival signaling, since inhibition of TNFR2 in winner cells attenuates cell competition. As tumor cells have also been described to harness TNFR2 for self-propagation, future work should investigate the role of TNFR2 in promoting cell competition in tumorigenesis, especially in tumor contexts with aberrant Bax expression. Furthermore, as we previously have not observed this extent of WT cell death in response to TNFα, we speculate that the presence of BaxKD cells may sensitize them to this stimulus. We believe this may potentially occur through chronic exposure along with the proximity and dosage of TNFα provided by BaxKD cells, thereby priming these cells for apoptosis. However, we have not investigated non-apoptotic responses of WT cells to winner cells that may be occurring in parallel and perhaps to a lesser extent. It would therefore be intriguing to investigate how these parameters may play a role in dictating cellular outcomes in response to TNFα stimulation.

It is additionally relevant to note that activation of p65-NF*κ*B signaling is highly context dependent, promoting cell death under one set of circumstances and cell survival and proliferation under another. The outcome therefore represents a culmination of diverse and often contradictory upstream events^78–80^. For example, it has been well established that activation of cIAP1/2 triggers activation of RIPK1 and TAK1 and degradation of I*κ*Bα, resulting in liberation and translocation of p65 to the nucleus where it induces target gene expression^41,81,82^. In addition to this, both cIAP1 expression and Smac deletion have been shown to induce NF*κ*B activation^42,83^. Nevertheless, additional work has also shown that depletion of cIAP1 as a result of increased MOMP promotes subsequent NIK activation in the absence of caspases, triggering NF*κ*B signaling and caspase-independent cell death (CICD)^84^. Although these and additional data explain how opposing signals can engage canonical and non-canonical effectors to promote NF*κ*B signaling, it remains to be fully elucidated how these signals are integrated to instruct cellular decisions. Nevertheless, it is known that factors such as degree of NF*κ*B activation, duration of signaling, dosage of external stimuli, relative abundance of promoting and antagonizing proteins, and overall environmental context all contribute to the final outcome^85,86^.

Functionally, we show that in vivo, Bax depletion results in tissue hypertrophy coupled with the observation of non-autonomous cell death surrounding Bax depleted cells. Furthermore, *BakcBax*^−/−^ mice exhibit enhanced tissue repair and regeneration following epidermal wound infliction. Similarly, skin and hair reconstitution assays revealed extensive generation of hairs in BaxKD versus WT HFSC derived grafts. Emphasizing the contribution of cell competition in vivo, BaxKD cells were overrepresented under mixed SC population conditions in comparison to WT cells in a manner attenuated by TNFα neutralization. We would like to note that while these grafts are derived of WT and BaxKD HFSCs, this does not rule out the possibility of competition occurring in the progeny of these SCs. Finally, grafts derived from SC populations including BaxKD HFSCs exhibited the formation of abnormal growths, indicating the potential of Bax depletion to contribute to pathological states. Future work is necessary to elucidate whether apoptotic dysregulation contributes to aberrant environmental remodeling via related mechanisms in other cellular contexts in vivo^10,87–90^. Collectively, these data demonstrate that apoptotic dysregulation can have critical effects on cellular and tissue homeostasis, with potentially profound implications for targeting cell competition-driven regeneration and tumorigenesis.

## Methods

### Mice

All animal studies were approved by the Committee on the Ethics of Animal Experiments of the Technion, Israel institute of Technology. Mice were purchased from the Jackson Laboratory. These mutant mice have targeted mutations of *Bax* and *Bak1*. For targeted mutation of *Bax*, Dr. Stanley J. Korsmeyer’s lab (Harvard Medical School) designed a vector to flank exons 2–4 with *loxP* sites. This construct was electroporated into 129 × 1/SvJ-derived RW4 embryonic stem (ES) cells.

Mice resulting from germline transmission of this *Bax*^*fl*^ allele (on a mixed B6;129 genetic background) were generated. For targeted mutation of *Bak1*, Dr. Craig B. Thompson’s lab (University of Pennsylvania) designed a vector to replace exons 3–6 (encoding the Bcl2 homology domains) with a neo cassette. This construct was electroporated into (129 × 1/SvJ x 129S1/Sv)F1-derived R1 ES cells. Mice resulting from germline transmission of this allele (on a mixed *B6;129* genetic background) were generated. These two mutant strains were bred together and maintained as homozygotes by Dr. Stanley J. Korsmeyer’s lab (Harvard Medical School) prior to arrival at The Jackson Laboratory. *Bak-Baxfl/fl* (*B6;129*-*Bax*^*tm2Sjk*^ *Bak1*^*tm1Thsn*^/*J*; JAX stock #006329), *K15-CrePGR* (*B6;SJL-Tg(Krt1-15-cre/PGR)22Cot/J*), and *ROSA-26-Confetti* (*B6.129P2-Gt (ROSA) 26Sortm1 (CAG-Brainbow2.1)Cle/J*) mice were purchased from Jackson Laboratories. *Foxn1*^*nu/nu*^ mice and *C57BL/6* black WT mice were purchased from Invigo.

Baxfl/fl mice were generated using standard homologous recombination floxing exon 6 surrounding the catalytic region QACGG of *Bax*. FRT flanked *Neo* cassette was inserted between the *LoxP* sites and FLP recombinase was used to remove the *Neo* cassette. Embryonic stem (ES) cells were targeted with the vector via electroporation. The screening of the ES cells was done by Genentech. *K15-CrePGR/Baxfl/fl* mice were prepared by crossing *K15-CrePGR* with *Baxfl/fl* mice. The promoter-specific expression of *CrePGR* (Cre fusion to progesterone receptor) enables conditional deletion of the *Bax* gene in the Krt15^+^ HFSC population, as well as controllable activation of CrePGR upon treatment with the synthetic PGR ligand, RU486. Only mice that were *Cre* positive were used for *K15-CrePGR* and *K15-CrePGR/Baxfl/fl* strains and only mice homozygous for the floxed and *R26* allele were used for *Baxfl/fl*. Harvested tissue samples were obtained from both female and male mice.

#### Cre-recombinase induction

Prior to induction with RU486, mice were shaved with electric clippers and treated topically with hair removal cream (Nair). To activate Cre-recombinase, RU486 was dissolved in DMSO (30 mg/mL) and diluted in PBS (1.5 mg/mL). A final concentration of 13.5 mg/kg was injected at the tip of the tail base, in conjunction with topical application (30 mg/ml RU486 in 80% ethanol). Injections were performed on 8-week-old telogenic mice for 5 consecutive days and skin was harvested 5 days post injection or 14 days post injection. Primers used for genotyping are outlined in Table S1.

#### Wound infliction

For wounding experiments, induction was performed as described above. At 10.5 weeks of age, full-thickness excision wounds (1.0 cm^2^) were inflicted on dorsal skin or 3 mm^2^ punch biopsy (Medex Supply) wounds on the tail skin. Wounds were monitored daily and wound size was measured using a transparent sheet. At harvesting timepoints, mice were sedated with isoflurane during injections and wounding and were administered Buprenorphine (0.1 mg/kg) prior to wounding for three days PWI. At the harvesting point, mice were euthanized with CO_2_ and the wounded skins were harvested and either embedded in OCT, paraffin or prepared for wholemounts.

#### Depilation

Three days prior to depilation (day −3), 8-week-old mice were subcutaneously injected with 200uL of BAI-1 (1 mg/kg)^91^ (MedChemExpress) along dorsal skin. Injections were continued daily for a total of five consecutive days (until day 2 post-depilation). On the day of depilation (day 0), dorsal hair of mice was trimmed with electrical clippers and wax strips were used to remove remaining hair from the dorsal skin. Mice were monitored daily for skin coloration changes. At day 9 post depilation, skin was harvested and embedded in OCT for further sectioning and histological staining.

#### Hair grafting

Grafting was performed based on protocols described^68–70^ with modifications. Briefly, silicon chambers were implanted onto the dorsal skin of female 8-week-old (P56) nude mice prior to grafting. For preparation of fresh neonatal dermal fibroblasts (DFs), WT (C57BL/6 black) pups were collected at P0–P2 and whole skin was removed and processed with dispase solution overnight at 4 °C. Dermis and epidermis were then separated, and the dermal layer was chopped and treated with collagenase I solution for 1 h at 37 °C for downstream processing as previously described^69^. In place of whole epidermal cell fractions, WT-mRFP1 and BaxKD-GFP HFSCs (up to passage 20) were expanded under standard HFSC culture conditions. Immediately prior to grafting, HFSCs were harvested through trypsinization for 10 min at 37 °C, followed by centrifugation for 5 min at 500xg. HFSCs and dermal cells were then counted and combined at a 1:3 ratio (~3 million HFSCs and ~9 million dermal cells per graft) in 1X sterile PBS at a volume of 150 μL per graft. Cell mixtures were then gently transplanted in a drop-wise manner into silicon chambers. Harvested cells were kept on ice for the entire duration of time. Silicon chambers were removed after 8–10 days, and grafts were collected 28 days after post-transplantation for downstream processing.

#### In vivo neutralization of TNFα

For grafting experiments, experimental groups were treated with either non-specific Rat LEAF purified IgGI (10 μg/g, Biolegend) or LEAF purified anti-mouse TNFα (10 μg/g). Antibodies were administered daily through direct injection onto grafting sites in 200 μL of sterile 1X PBS from days 0-4 post-grafting.

### Cloning of lentiviral vectors for RNA silencing and overexpression

To generate lentiviral vectors, we used an H2B-GFP empty PLKO.1 lentiviral vector suitable for silencing (Addgene #25999). We next digested the PLKO.1 lentiviral vector with RsrII and EcoRI enzymes. Synthesized shRNA oligonucleotide target sequences (IDT) were annealed and ligated into the digested PLKO.1 vector. Knockdown targeting sequences were designed by Sigma as outlined in Table S1 (Supplementary Information). The pHIV-EGFP plasmid, a gift from Bryan Welm & Zena Werb (Addgene plasmid# 21373)^92^, was used as a backbone for gene overexpression. The backbone lentiviral vector was digested using SmaI HF (NEB) enzyme. The Bcl2 gene was amplified from mouse HFSC cDNA using primers with adaptors for Gibson Assembly (Table S1), and amplicons were run on a 2% agarose gel and extracted using the QIAquick Gel Extraction kit (Qiagen). Amplicons were then ligated into the backbone using the Gibson Assembly Master Mix (NEB).

Ligated vectors were transformed into chemically competent Stable3 cells and cultures were grown on agar plates with Carbenicillin (50 μg/mL) overnight at 37 °C. Individual clones were picked and grown overnight in standard Luria Broth (LB) at 37 °C, and plasmids were extracted using the Qiagen miniprep kit. Samples were sent for standard sequencing through Macrogen Europe. Positive clones were expanded overnight at 37 °C and plasmids were harvested using the PureYield Plasmid Maxi Prep kit (Promega).

### Viral production

For viral production, HEK293FT cells (passage 10 or under) were grown in high glucose DMEM media supplemented with 10% fetal bovine serum (FBS), 1% Pen/Strep, 1% L-glutamine, 1% Sodium Pyruvate, 1% Sodium Bicarbonate, and 1% non-essential amino acids (NEAA). Prior to transfection, cells were grown to 85% confluence and passaged 1 day prior onto poly-L-lysine (PLL)-coated 15 cm dishes.

Cells were transfected with the lentiviral target vector together with 2^nd^ generation viral packaging vectors using 1 mg/mL polyethylenimine (PEI) transfection reagent at 1:1.5 DNA:PEI ratio and incubated overnight in starvation media. The following morning, media was changed to standard growth media (as described above) and incubated overnight. Day 1 of viral-containing media was collected the following morning and media was replaced. This was repeated for Day 2 of collection. Viral media was concentrated using Amicon Ultra Centrifugal filtration units (Millipore), aliquoted, and stored at −80 °C until further use.

### Generation of stable cell lines

HFSCs at 60% confluence were transduced with virus using polybrene reagent at 1:1000 (10 mg/mL stock) and incubated overnight in standard media. 72 h post-transduction, transduced cells were isolated based on fluorescence expression using FACS.

### RNA extraction, reverse transcription, and real-time quantitative PCR (RT qPCR)

RNA was isolated using TRIzol (Sigma) and cDNA was synthesized using up to 1ug of RNA (qScript cDNA Synthesis Kit). Real-time quantitative PCR (RT qPCR) was carried out on the CFX Connect Real Time PCR Detection System (BioRad) with the PerfeCTa SYBR Green FastMix (Quanta), with gene-specific primers outlined in Table S1. Amplicon levels were analyzed in triplicates and quantified relative to a standard curve comprising cDNA. Values were normalized to housekeeping gene levels (RPLP0 or GAPDH). All experiments were performed in biological and technical triplicates. Reactions were: 3 min at 95 °C, 40 cycles of 10 s at 95 °C and 30 s at 60 °C with addition of a melt curve step: 10 s at 95 °C, and increments of 0.5 °C every 5 s between 65 °C and 95 °C. Fold change in gene expression was calculated after normalization of target to housekeeping genes, and comparison between conditions using the delta delta Ct method.

### Histology

Isolated dorsal wounds at 18 or 30 days PWI were fixed for 2 h in 4% paraformaldehyde (PFA). Wounds were embedded in paraffin in the following conditions: 80% EtOH (45 min), 95% EtOH (45 min x2), 100% EtOH (45 min x2), 1:1 EtOH:xylene (45 min), xylene (45 min), xylene (30 min), paraffin (45 min, 54 °C, X3) and samples were then embedded in paraffin. Deparaffinization was achieved in the following conditions: xylene (5 min, x2), 100% EtOH (2 min, x3), 95% EtOH (2 min), H_2_O wash. For histological analysis of depilation and grafting experiments, skin samples were harvested and fixed as described above, followed by embedding into OCT for preparation of cryosections. 

For Hematoxylin and Eosin staining, deparaffinized or cryopreserved 20μm sections were treated with: Hematoxylin (1 min; Sigma HHs32), H_2_O rinse, differentiator (0.3% alcoholic HCl, 2 dips), H_2_O rinse, 95% EtOH (1 min), Eosin (Sigma, HT110116, 2 min), 95% EtOH (10 s), 100 EtOH (1 min, x2), xylene (1 min, x3).

### Immunofluorescence

HFSCs were grown on PLL-coated glass coverslips for downstream analysis. Samples were fixed in 4% PFA at room temperature (RT) for 30 min. For wounded skin sections, harvested skin was embedded in either OCT or paraffin. For OCT sections, 15 μm sections were mounted onto microscopy slides and fixed in 4% PFA at RT for 30 min. For paraffin sections, 7 μm sections were mounted onto microscopy slides and deparaffinization was achieved in the following conditions: xylene (5 min, x2), 100% EtOH (2 min, x3), 95% EtOH (2 min), H_2_O wash.

For preparation of wholemount samples, isolated tail and dorsal tissue were treated with 20 mM EDTA for 4 h (tail) and 6 h (dorsal) at 37 °C for efficient separation of the epidermis from the dermis. Following separation, samples were fixed in 4% PFA for 2 h at RT.

After fixation or deparaffinization, all samples were washed in 1X PBS (5 min, x2) and blocked for 2 h in blocking buffer consisting of 10% goat serum, 2% BSA, 0.2% Triton-X. Primary antibodies were diluted in blocking buffer and cells or tissues were incubated overnight at 4 °C. Samples were washed at least three times with 1X PBS. Secondary antibodies were incubated for 1 h at RT followed by 4 washes with 1X PBS. The following primary antibodies were used: Bax (Mouse, 1:100, Thermo Scientific, cat. #MA5-14003, lot #QK2110159; 1:100, Cell Signaling, cat. #5023), cleaved Caspase-3 (Rabbit, 1:100, Cell Signaling, cat. #9661 S, lot #47), cleaved Caspase-8 (Rabbit, 1:100, Cell Signaling, cat. #8592), Ki67 (rat, 1:100, eBioscience: cat. #14-5698-82, lot # 2196796), TNFα (Mouse, 1:100, abcam, cat. #1793, lot #GR2370127-1), cIAP1 (Rabbit, 1:100, Santa Cruz, cat. #7943), NFkB p65 (Mouse, 1:100, Santa Cruz #8008), Keratin-15 (Mouse, 1:100, Abcam, cat. #ab80522), and CD34 (Rat, 1:100, Pharmingen, cat. #553731).

Antibody staining was visualized using secondary antibodies conjugated to Alexa Fluor dyes: 488, 546, and 633 antibodies (1:250, Life Technologies, cat. #s: A11001, A11003, A11006, A11008, A11010, A11039, A11040, A11081, A21103, A21050, A21070, A21094). Additional stains were performed using Terminal deoxynucleotidyl transferase dUTP nick end labeling (TUNEL; ApopTag TdT kit, Millipore) and mitochondrial labeling MitoTracker Red CMXRos (ThermoFisher). Analyses were performed on a Zeiss LSM-880 confocal microscope.

### Protein extraction and immunoblotting

For whole cell lysate extraction, cells were washed with ice-cold 1XPBS and collected by scraping on ice, followed by centrifugation (1800x *g*, 5 min at 4 °C). Cell pellets were lysed using RIPA lysis buffer containing protease inhibitor cocktail (1:200, Abcam) and phosphatase inhibitor cocktail (1:100, Sigma), followed by incubation on ice for 30 min and centrifugation (22,000 x g, 15 min at 4 °C). The protein-containing supernatants were collected for downstream processing. For nuclear fractionation, cells were washed with ice-hold 1XPBS and collected by scraping on ice and centrifuged at 500 x g for 3 min. Downstream steps were performed using the NE-PER Nuclear and Cytoplasmic Extraction kit (ThermoFisher) according to manufacturer protocols.

Protein concentration was measured (Bradford reagent, BioRad). Protein samples were denatured through addition of 5X LSB containing β-mercaptoethanol and 5-minute incubation at 95 °C. Protein samples were resolved by running on 12.5 % SDS-PAGE and electrotransferred to a nitrocellulose membrane (Whatman). Transfer of proteins was visualized using Ponceau Red dye (Sigma). Membranes were blocked in 5% dry skimmed milk or 3% BSA in TBS-T for 1 h at RT and incubated with primary antibodies (1:1000) overnight. Membranes were then incubated with secondary antibody (ms-HRP or rb-HRP, 1:10,000) for 1 h at RT, washed 3X with TBS-T for a minimum of 10 min each, and chemiluminescence was visualized using the iBright Imaging Systems (Invitrogen). Densitometric values were obtained through normalization of proteins of interest using either the iBright Imaging Systems or ImageJ V.1.53. The following antibodies were used: Bax (1:1000, Cell Signaling, cat. #5023), cleaved Parp1 (1:1000, Cell Signaling, cat. #9544), cleaved Caspase-3 (1:1000, Cell Signaling, cat. #9661 S, lot #47), GAPDH (1:10000, Sigma, cat. #G9545, lot #128M4817V), α-tubulin (; 1:10000, Santa Cruz, cat. #23948, lot #C0415), b-actin (1:10000, Santa Cruz, cat. #81178, lot #J1116), b-tubulin (1:10000, Cell Signaling, cat. #2128), Histone-3 (1:10000, Abcam, cat. #18521; 1:10000, Cell Signaling, cat. #4499), cIAP1 (Santa Cruz, Cat. #7943), cIAP2 (Santa Cruz, cat. #sc-7944), IkBa (1:1000, Santa Cruz, #1643), NFkB p65 (1:1000, Santa Cruz cat. #8008, lot #H1819; 1:1000, Cell Signaling #8242), and TNFR2 (1:1000, Abcam, cat. #15563).

### Flow cytometry

#### Cell competition assays

Co-cultured HFSCs were isolated using either fluorescent activated cell sorting (FACS) performed on a BD FACSAria IIIu for downstream RNA processing, or analyzed on a BD LSR I or Attune NxT flow cytometer. Cells were collected at distinct timepoints to quantify changes in cell population ratios between 0 and 10 DPS.

#### Quantification of CD34^+^ HFSC populations in vivo

Epidermal cell populations were isolated from telogenic dorsal skin of mice, and HFSC populations were subjected to immunostaining as previously described^28,29^. Briefly, single cell suspensions of epidermal cells from control or RU486-induced *BakcBax*^−/−^ mice were were obtained after digestion with trypsin and filtration, followed by immunostaining against the following antibodies: Integrin-a6 PE (1:200, eBioscience, Cat. #12-0495-82), ScaI FITC (1:200, eBioscience, Cat. #553335), and CD34 eFluor 660 (1:100, eBioscience, Cat. #50-0341-82), alongside DAPI (1:10,000). Cells were incubated with antibodies diluted in staining buffer (3% chelated FBS) on ice for 30 min, followed by washing and centrifugation steps at 500x *g* for 5 min at 4 °C before FACS analysis (pertaining to Fig. 6k). *n* = 3 mice were pooled for each condition. The CD34^+^ population is represented as a percentage from the parental Integrin-α6^+^/Sca1^-^ epidermal pool.

Downstream analysis of FACS data was performed using FCS Express v.7.0, FlowJo v.10.9, BDFACS Diva v.9.0. For gating strategy, please see Supplementary Fig. 11.

### Cell culture

#### Cell maintenance

Feeder free isolated a6^+^ScaI^-^CD34^+^ HFSCs were isolated and grown as previously described^28^. HFSC medium was prepared using Dulbecco’s modified Eagle medium (DMEM)/F12 3:1 (Biological Industries) containing L-glutamine (Biological Industries; 1:100), penicillin/streptomycin (Biological Industries; 1:100), 10% chelated fetal bovine serum, 5 μg/ml insulin (Sigma I-5500), 5 μg/ml transferrin (Sigma T-2252), 2 × 10^−12^M T3 (3,30-triiodo-L-thyronine; Sigma T-2752), 400 ng/ml hydrocortisone (Sigma H0888), cholera toxin (10^−10^M), and CaCl_2_ (50 μM).

Media was replaced every 2 days and cells were grown to 80–85% confluence prior to passaging. Cells were passaged by incubating with Trypsin for 10 min followed by centrifugation at 500 x g for 5 min.

Cellular treatments were performed as described below. Cell lines were passage matched across all experiments. Media from cells underwent routine testing to ensure culturing under mycoplasma-free conditions.

#### Apoptotic induction assays

Cells were treated with ABT199 (Bcl-2 inhibitor) (50 μM) or TNFα (20 ng/mL) + cycloheximide (CHX) (protein synthesis inhibitor) (10 μg/mL) immediately prior to acquisition of initial brightfield (BF) images. BF images and samples were taken every 30 min for up to 4 h for ABT199 treatment and up to 6 h for TNFα + CHX treatment for downstream processing. The percentage of live cells were analyzed at each timepoint using Trypan Blue to determine cell survival. MitoTracker Red CMXRos (200 nM, ThermoFisher) was used to visualize mitochondria during ABT199 treatment. Untreated cells were visualized under the same conditions.

#### Colony formation assay (CFA)

Cells were seeded at low density (1,000 cells per well of a 6-well plate) and media was changed every two days. Cells were monitored daily and colony size and growth were measured at specified time points between 1 and 8 days post seeding (DPS). For histology, colonies were fixed with 4% PFA for 15 min at RT, followed by washing with 1X PBS. Toluidine Blue O dye (Acros Organics) (0.1% in ddH_2_O) was then directly added to wells and incubated for 20 min at RT, followed by several washes with ddH_2_O prior to image acquisition.

#### Cell competition co-culture assays

Cell populations specified in each experiment were counted, mixed at 1:1 cell ratio, and seeded in co-culture (eg., WT and BaxKD co-cultures alongside WT only and BaxKD only control cultures; WT and BaxKD-TNFαKD co-cultures alongside WT and BaxKD control co-cultures, etc.). Co-cultures were monitored daily and maintained under standard growth conditions as described above (unless otherwise noted, such as in the case of treatments) for the duration of the experiment (up to 10DPS). For live imaging experiments, co-cultures were grown in a standard incubator (37 °C, 5% CO_2_) until substantial cell-cell contact was observed, after which culture plates were transferred into an incubation chamber for live imaging under the same conditions for the specified duration of time. For co-culture experiments requiring additional treatments or other downstream analysis (eg., FACS, RNA extraction, in situ image analysis, TNFα neutralization, TNFα stimulation, etc.), cells were processed as described for each method.

#### BAI-1 chemical inhibition

Cells were treated with BAI-1 (100 μM)^91^ (MedChemExpress) and samples were harvested at timepoints including 15, 30, 45, 60, and 90 min alongside vehicle (NT) controls for downstream protein, RNA, and IF assays. All experiments were performed under the same experimental conditions.

#### TNFα stimulation

Both WT and BaxKD SCs were incubated in starvation media for 12 h prior to stimulation, in order to synchronize cell cycles^93^. TNFα (20 ng/mL) was added into media, and samples were visualized or harvested for downstream analysis at specified timepoints. All experiments were performed under the same experimental conditions.

#### Conditioned media assays

In the first assay (pertaining to Supplementary Fig. 2e), cells were seeded at low confluency (20%) and treated daily with 50% conditioned media (CM) from WT or BaxKD cells for 5 days. CM was harvested from high confluence (80%) WT or BaxKD cells. Cells were monitored daily for morphological changes characteristic of apoptosis. In the second assay (pertaining to Supplementary Fig. 2f, g), CM was harvested from high confluence (80%) WT cells, BaxKD cells, or competitive co-culture cells. Next, individual WT or BaxKD cells were treated for up to 24 h with each type of CM and harvested at various timepoints as previously described^34^. Downstream immunofluorescent analysis of cCp8 expression in treated cells was used as an indicator of extrinsic death induction.

#### TNFα neutralization in vitro

Competitive WT and BaxKD co-cultures were seeded at equal ratios in media containing the TNFα inhibitor Etanercept (20 μg/mL)^94^ (Sigma) or vehicle (NT). Cultures were harvested at 0DPS, 1DPS, and 3DPS for FACS quantification of population ratios.

### Live imaging

Imaging of cell competition assays was performed at 5DPS, and proliferation at 3DPS using a ZEISS inverted microscope system containing an incubator for optimal cell growth conditions (37 °C, 5% CO_2_). Cells were visualized using endogenous fluorescence together with BF imaging for a duration of 12–24 h.

### In situ analysis of cell competition

In situ analysis of cell competition was performed by harvesting competitive co-cultures at various timepoints after seeding (in parallel to FACS analysis), followed by image analysis using a fluorescent microscope and quantification of the number of cells pertaining to each cell population via ImageJ v.1.53.

### RNA sequencing data analysis

SC RNA-seq data from Haensel et al. (GEO #GSE142471)^19^ was analyzed in RStudio v.1.3.1093 using the Seurat package v.3.2.2. After identification of a HFSC bulge cell cluster based on canonical marker expression, CD34^+^Krt15^+^ expressing cells were subset and assessed for expression of various genes within this population.

### Quantification and data analysis

Data are mean ± SEM. The *n* values represent biological repeats measured independently as specified in each figure legend. Each experiment included at least three biological replicates and three technical replicates where appropriate, and was repeated a minimum of two times. Significance was determined by performing parametric unpaired two-tailed Student’s t-test, where *p < 0.05, **p < 0.01 and ***p < 0.001. Analysis software includes: ImageJ v.1.53, ZEN v.3.0, and iBright Imaging Systems programs.

#### Image-based analyses

Identical parameters were utilized for analysis of photos within the same experiment using ZEN v.3.0 (Carl Zeiss Microscopy) and/or ImageJ v.1.53.

#### RNA sequencing analyses

Analyses derived from single cell RNA-sequencing dataset(s) specified were performed using Seurat v.3.2.2. in RStudio v.1.3.1093.

### Statistics and reproducibility

All experiments were repeated at least twice with similar results obtained. Each independent experiment included at least three biological repeats unless otherwise indicated in the figure legends. No statistical methods were used to predetermine sample sizes. Data distribution was assumed to be normal, but this was not formally tested. Data collection and analysis were not performed blind to the conditions of the experiments. No animals or data points were excluded from the analyses. Data are presented as mean ± s.e.m. Statistical significance was determined by unpaired two-tailed Student’s *t*-test, where *P < 0.05, **P < 0.01 and ***P < 0.001. The following software were used: Excel v.16.19 and GraphPad Prism v.9.5.1.

### Reporting summary

Further information on research design is available in the Nature Portfolio Reporting Summary linked to this article.

### Supplementary information

Supplementary Information

Description of Additional Supplementary Files

Supplementary Movie 1

Supplementary Movie 2

Reporting Summary

### Source data

Source Data

## Data Availability

Further data supporting the findings of this study are available from the corresponding author upon reasonable request. Source data are provided with this paper.
