# Supplementary material for: Apoptotic dysregulation mediates stem cell competition and tissue regeneration
Source: Nat Commun. 2023 Nov 20;14:7547. doi: 10.1038/s41467-023-41684-x (PMC10662150; doi:10.1038/s41467-023-41684-x)
Supplement: Supplementary file 1 — Supplementary Information [file 41467_2023_41684_MOESM1_ESM.pdf]

Supplemental Information

Apoptotic Dysregulation Mediates Stem Cell Competition and Tissue Regeneration

Marianna Yusupova[1], Roi Ankawa[1,2], Yahav Yosefzon[1], David Meiri[1], Ido Bachelet[2] and Yaron Fuchs[1,2*]

[1] Faculty of Biology, Technion-Israel Institute of Technology, Haifa, Israel.
[2] Augmanity, Rehovot, Israel.
***Corresponding Author**: Yaron Fuchs; yaronfox@gmail.com

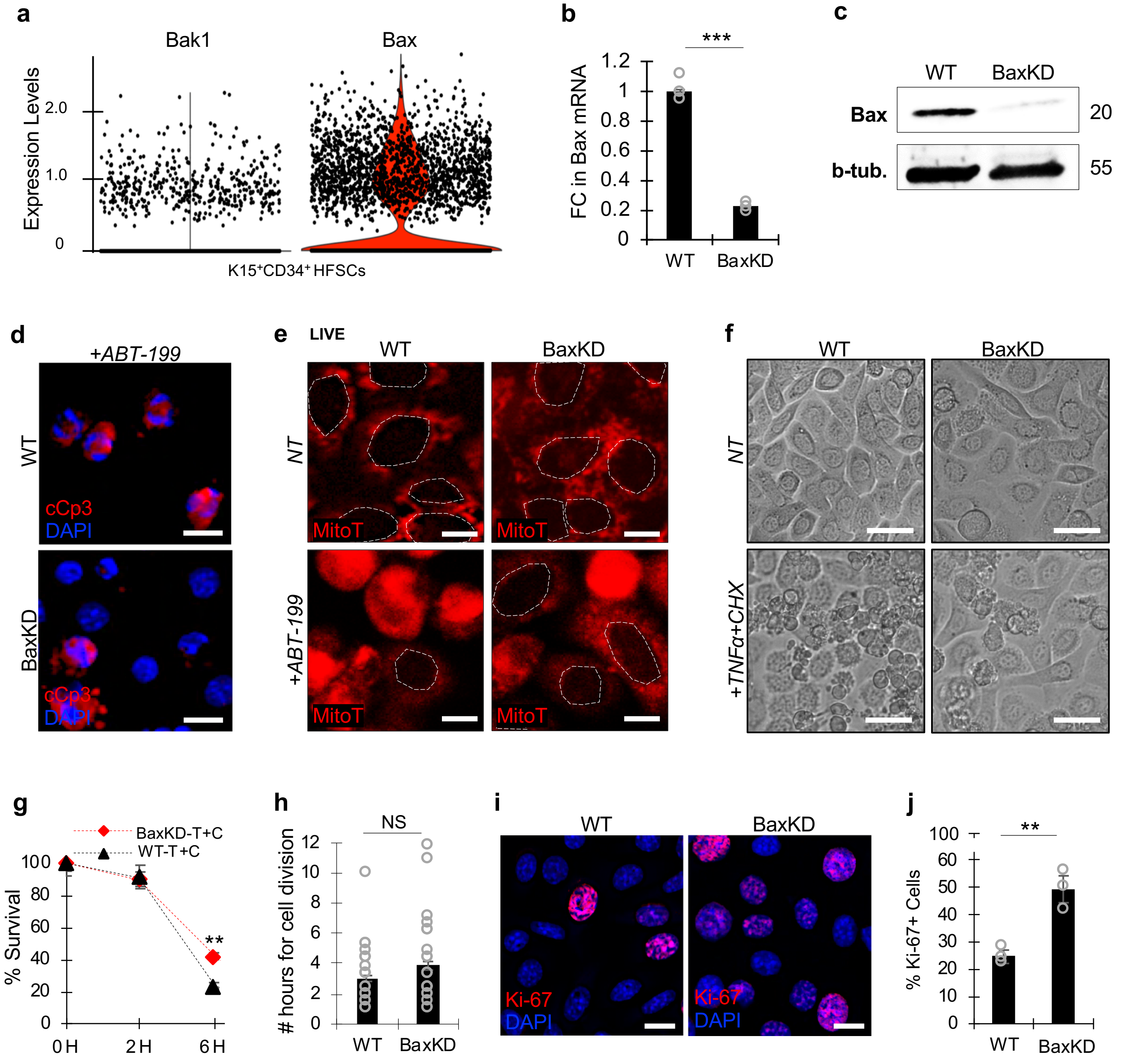

a
Bak1
Bax
Expression Levels
2.0
1.0
0
K15+CD34+ HFSCs
b
***
FC in Bax mRNA
1.2
1
0.8
0.6
0.4
0.2
0
WT
BaxKD
c
WT
BaxKD
Bax
20
b-tub.
55
d
+ABT-199
WT
cCp3
DAPI
BaxKD
cCp3
DAPI
e
LIVE
WT
BaxKD
NT
MitoT
MitoT
+ABT-199
MitoT
MitoT
f
WT
BaxKD
NT
+TNFα+CHX
g
BaxKD-T+C
WT-T+C
% Survival
100
80
60
40
20
0
**
0 H
2 H
6 H
h
NS
hours for cell division
12
10
8
6
4
2
0
WT
BaxKD
i
WT
BaxKD
Ki-67
DAPI
Ki-67
DAPI
j
**
% Ki-67+ Cells
100
60
50
40
30
20
10
0
WT
BaxKD

Supplementary Figure 1. Bax depletion confers enhanced apoptotic resistance and proliferation.

a Violin plots from single-cell RNA-seq data depicting single cell distributions of Bak1 (left) and Bax (right) expression in the HFSC population. Each point represents an individual cell.

b FC in Bax mRNA levels in WT versus BaxKD HFSCs.

c IB against Bax (top) and β-tubulin (bottom) in WT and BaxKD cells.

d IF staining against cCp3 in WT (top) and BaxKD (bottom) HFSCs treated with ABT199 after 4 h.

e Live fluorescent imaging of ABT199 treated WT (left) or BaxKD (right) HFSCs visualized with MitoTracker (MitoT). Dotted lines mark the nuclear boundary.

f BF images of WT (left) or BaxKD (right) cells treated with TNFα + cyclohexamide (CHX) after 6 h.

g Percentage of surviving cells after TNFα+CHX (T+C) treatment based on (**f**).

h Quantification of number of hours required for cell division completion during live imaging in WT and BaxKD HFSC cultures.

i IF staining against Ki-67 in WT and BaxKD cells.

j Percentage of Ki-67$^+$ cells based on (**i**).

Scale bar represents 20μm for **d, e, i**; 50μm for **f**; 100μm for **h**. *n* = 3 biological replicates per condition per experiment unless otherwise specified. Each experiment was repeated at least 2 times with similar results. All data are mean and ± SEM. P-values were determined by unpaired two-tailed t-test. P-value <0.05*, <0.01**, <0.001***.

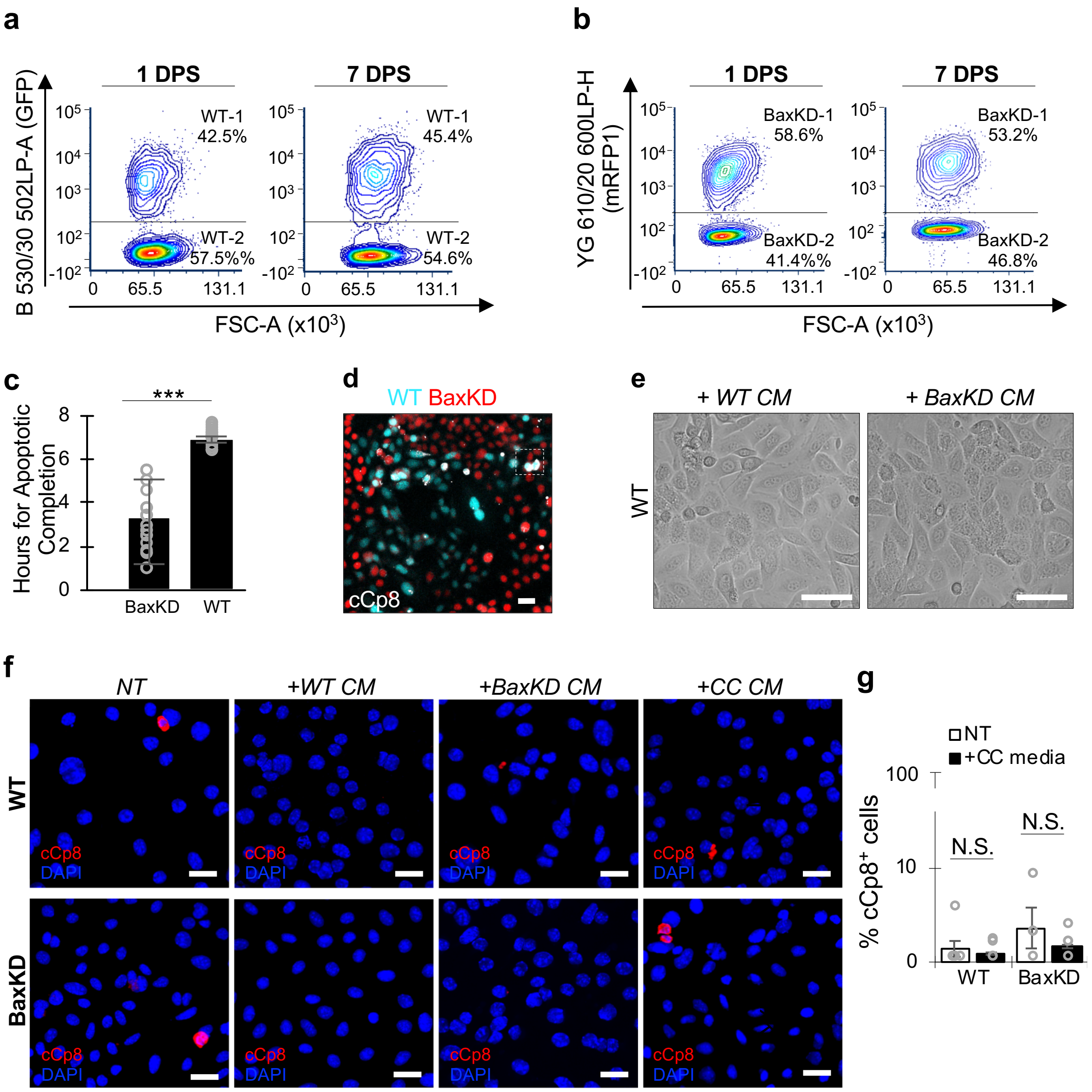
a
1 DPS
7 DPS
B 530/30 502LP-A (GFP)
WT-1
42.5%
WT-2
57.5%%
WT-1
45.4%
WT-2
54.6%
FSC-A (x10³)
b
1 DPS
7 DPS
YG 610/20 600LP-H (mRFP1)
BaxKD-1
58.6%
BaxKD-2
41.4%%
BaxKD-1
53.2%
BaxKD-2
46.8%
FSC-A (x10³)
c
Hours for Apoptotic Completion
***
BaxKD
WT
d
WT BaxKD
cCp8
e
+ WT CM
+ BaxKD CM
WT
f
NT
+WT CM
+BaxKD CM
+CC CM
WT
BaxKD
cCp8
DAPI
g
NT
+CC media
% cCp8⁺ cells
N.S.
N.S.
WT
BaxKD

Supplementary Figure 2. Bax-depleted cells actively eliminate WT neighbors in a contact-dependent manner.

a, b FACS plots depicting percentages of WT populations (WT-1 and WT-2) (**a**) and BaxKD populations (**b**) in co-culture 1 and 7 DPS.

c Graph depicting the number of hours until apoptotic completion was observed by WT cells in the vicinity of BaxKD cells (left column) versus other WT cells (right column) after observed contact (based on Figure 2f). More than 50 apoptotic cells evaluated across *n* = 3 randomly selected fields and *n* = 3 biological replicates.

d IF staining against cleaved Caspase 8 (cCp8) in WT and BaxKD co-cultures 7DPS. Inset represents region depicted in Figure 2j.

e BF images depicting WT HFSCs treated with conditioned media (CM) harvested from WT or BaxKD HFSCs.

f IF staining against cleaved Caspase 8 (cCp8) in WT (top) or BaxKD (bottom) cells after 24-hour treatment with CM from WT only, BaxKD only, or WT+BaxKD cell competition (CC) HFSC co-cultures versus vehicle control (NT).

g Quantification of percentage of $cCp8^+$ HFSCs treated with CC CM or NT after 24 hours based on (**f**) (rightmost panels). At least 250 cells across *n* = 4 biological replicates evaluated per condition.

Scale bar represents 20μm in **d**; 50μm in **e**; 25μm in **f**. *n* = 3 biological replicates per condition per experiment unless otherwise specified. Each experiment was repeated at least 2 times with similar results. All data are mean and ± SEM. P-values were determined by unpaired two-tailed t-test. P-value <0.05*, <0.01**, <0.001***.

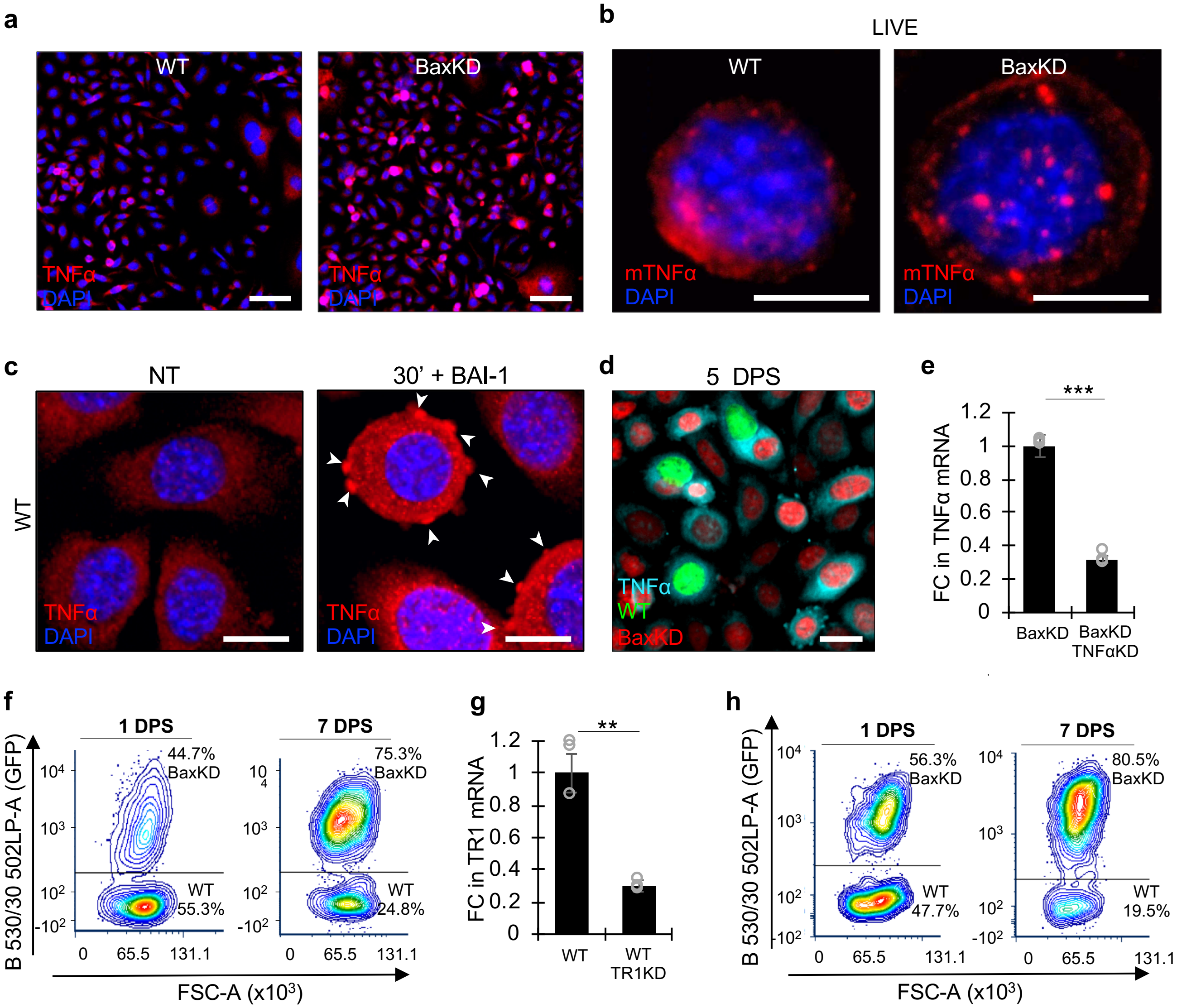

a
WT
BaxKD
TNFα
DAPI
b
LIVE
WT
BaxKD
mTNFα
DAPI
c
NT
30' + BAI-1
WT
TNFα
DAPI
d
5 DPS
TNFα
WT
BaxKD
e
***
FC in TNFα mRNA
1.2
1
0.8
0.6
0.4
0.2
0
BaxKD
BaxKD TNFαKD
f
1 DPS
7 DPS
44.7% BaxKD
WT 55.3%
75.3% BaxKD
WT 24.8%
B 530/30 502LP-A (GFP)
FSC-A (x10³)
0
65.5
131.1
g
**
FC in TR1 mRNA
WT
WT TR1KD
h
1 DPS
7 DPS
56.3% BaxKD
WT 47.7%
80.5% BaxKD
WT 19.5%
B 530/30 502LP-A (GFP)
FSC-A (x10³)

Supplementary Figure 3. Winner cells harness TNFα for elimination of loser cells.

a IF staining against TNFα in WT and BaxKD HFSCs.

b IF staining against TNFα in live non-permeabilized WT and BaxKD HFSCs.

c IF staining against TNFα in WT HFSCs after 30 min of vehicle no treatment (NT) (left) or BAI-1 treatment (right). Arrowheads denote membranal “hubs.”

d IF staining against TNFα (cyan) co-cultures of WT (green) and BaxKD (red) cells 5DPS.

e FC in mRNA levels of TNFα in BaxKD versus BaxKD+TNFαKD HFSCs.

f FACS plot for control cell competition condition pertaining to Figure 3h-i depicting percentage of BaxKD and WT HFSCs in co-cultures 1 and 7DPS.

g FC in mRNA levels of TNFR1 in BaxKD versus BaxKD+TNFR1KD HFSCs.

h FACS plot for control cell competition condition pertaining to Figure 3j-k depicting percentage of BaxKD and WT HFSCs in co-cultures 1 and 7DPS.

Scale bar represents 100μm in **a**; 10μm in **b, c** 20μm in **d**. *n* = 3 biological replicates per condition per experiment unless otherwise specified. Each experiment was repeated at least 2 times with similar results. All data are mean and ± SEM. P-values were determined by unpaired two-tailed t-test. P-value <0.05*, <0.01**, <0.001***.

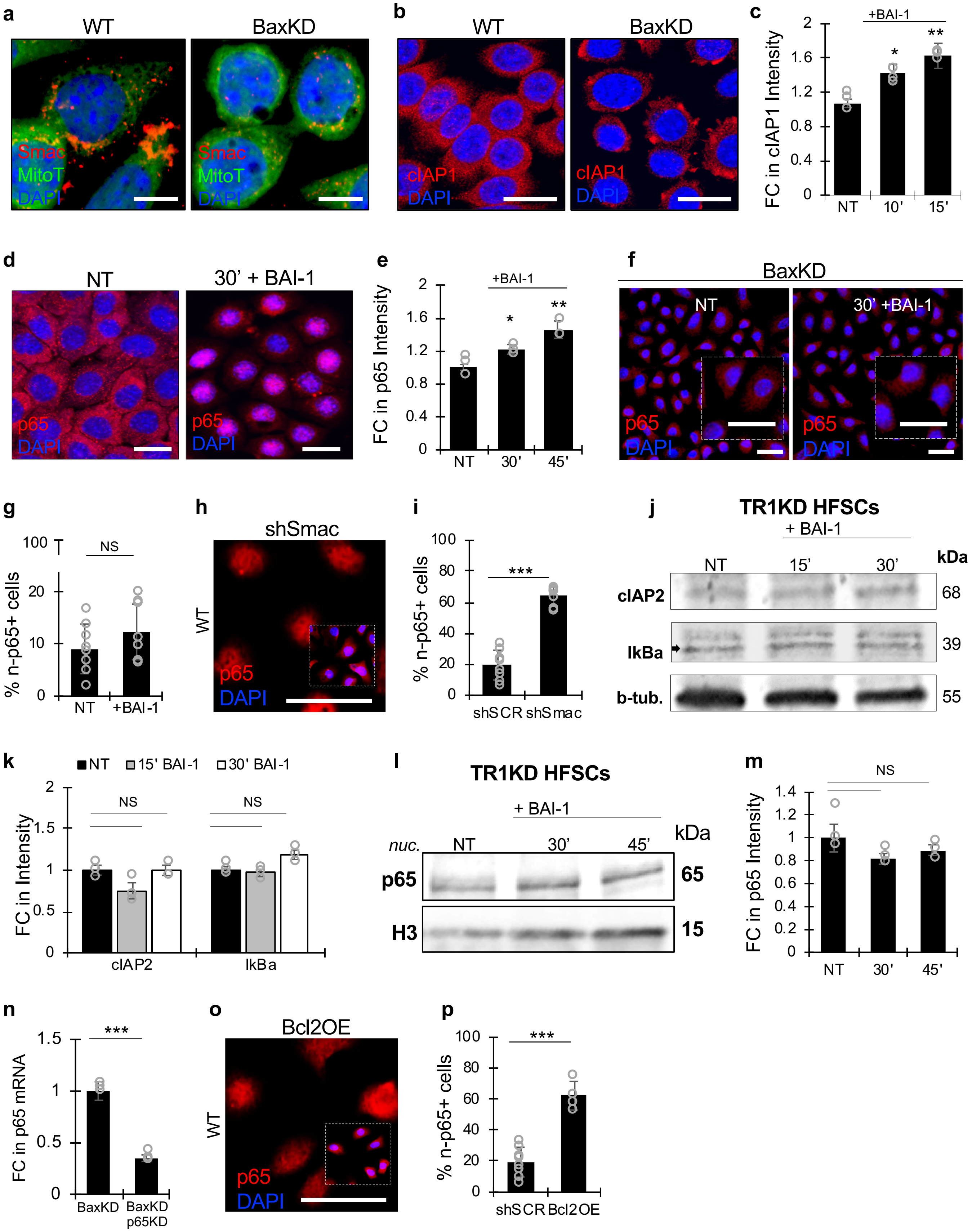
a
WT
BaxKD
Smac
MitoT
DAPI
b
WT
BaxKD
cIAP1
DAPI
c
+BAI-1
FC in cIAP1 Intensity
2
1.6
1.2
0.8
0.4
0
*
**
NT
10'
15'
d
NT
30' + BAI-1
p65
DAPI
e
+BAI-1
FC in p65 Intensity
2
1.6
1.2
0.8
0.4
0
*
**
NT
30'
45'
f
BaxKD
NT
30' +BAI-1
p65
DAPI
g
100
20
10
0
NS
% n-p65+ cells
NT
+BAI-1
h
shSmac
WT
p65
DAPI
i
100
80
60
40
20
0
***
% n-p65+ cells
shSCR
shSmac
j
TR1KD HFSCs
+ BAI-1
NT
15'
30'
kDa
cIAP2
68
IkBa
39
b-tub.
55
k
NT
15' BAI-1
30' BAI-1
FC in Intensity
2
1.5
1
0.5
0
NS
NS
cIAP2
IkBa
l
TR1KD HFSCs
+ BAI-1
kDa
nuc.
NT
30'
45'
p65
65
H3
15
m
NS
FC in p65 Intensity
1.4
1.2
1
0.8
0.6
0.4
0.2
0
NT
30'
45'
n
FC in p65 mRNA
1.5
1
0.5
0
***
BaxKD
BaxKD p65KD
o
Bcl2OE
WT
p65
DAPI
p
100
80
60
40
20
0
***
% n-p65+ cells
shSCR
Bcl2OE

Supplementary Figure 4. Bax inhibition results in activation of p65.

a, b IF staining against Smac together with MitoTracker dye (**a**) or IF staining against cIAP1 (**b**) in WT versus BaxKD HFSCs.

c Densitometric quantification of cIAP1 protein levels based on Figure 4d.

d IF staining against p65 in WT HFSCs after 30 min of BAI-1 treatment or NT.

e Densitometric quantification of nuclear p65 protein levels based on Figure 4h.

f IF staining against p65 in BAI-1 versus NT control treated BaxKD cells.

g Quantification of percentage of cells with nuclear p65 translocation based on (**f**).

h IF staining against p65 in WT HFSCs 24 hours post-transfection with shSmac (left).

i Quantification of percentage of cells with nuclear p65 translocation based on (**h**).

j IB against cIAP2 (top), IkBa (middle), and β-tubulin (bottom) in TNFα receptor 1 knockdown (TR1KD) cells treated with BAI-1 after 15 and 30 min versus NT control.

k Densitometric quantification of cIAP2 and Ikba proteins levels based on (**j**).

l IB against p65 and H3 in the nuclear fractions of TR1KD HFSCs NT, 30, and 45 min after BAI-1 treatment.

m Densitometric quantification of nuclear p65 protein levels based on (**l**).

n FC of p65 mRNA in BaxKD versus BaxKD+p65KD HFSCs.

o IF staining against p65 in WT HFSCs 24 h post-transfection with Bcl2 overexpression (Bcl2OE) vector.

p Quantification of percentage of cells with nuclear p65 translocation based on (**o**).

Scale bar represents 15μm in **a**; 30μm in **b, d**; 50μm in **f, h, o**. *n* = 3 biological replicates per condition per experiment unless otherwise specified. Each experiment was repeated at least 2 times with similar results. All data are mean and ± SEM. P-values were determined by unpaired two-tailed t-test. P-value <0.05*, <0.01**, <0.001***.

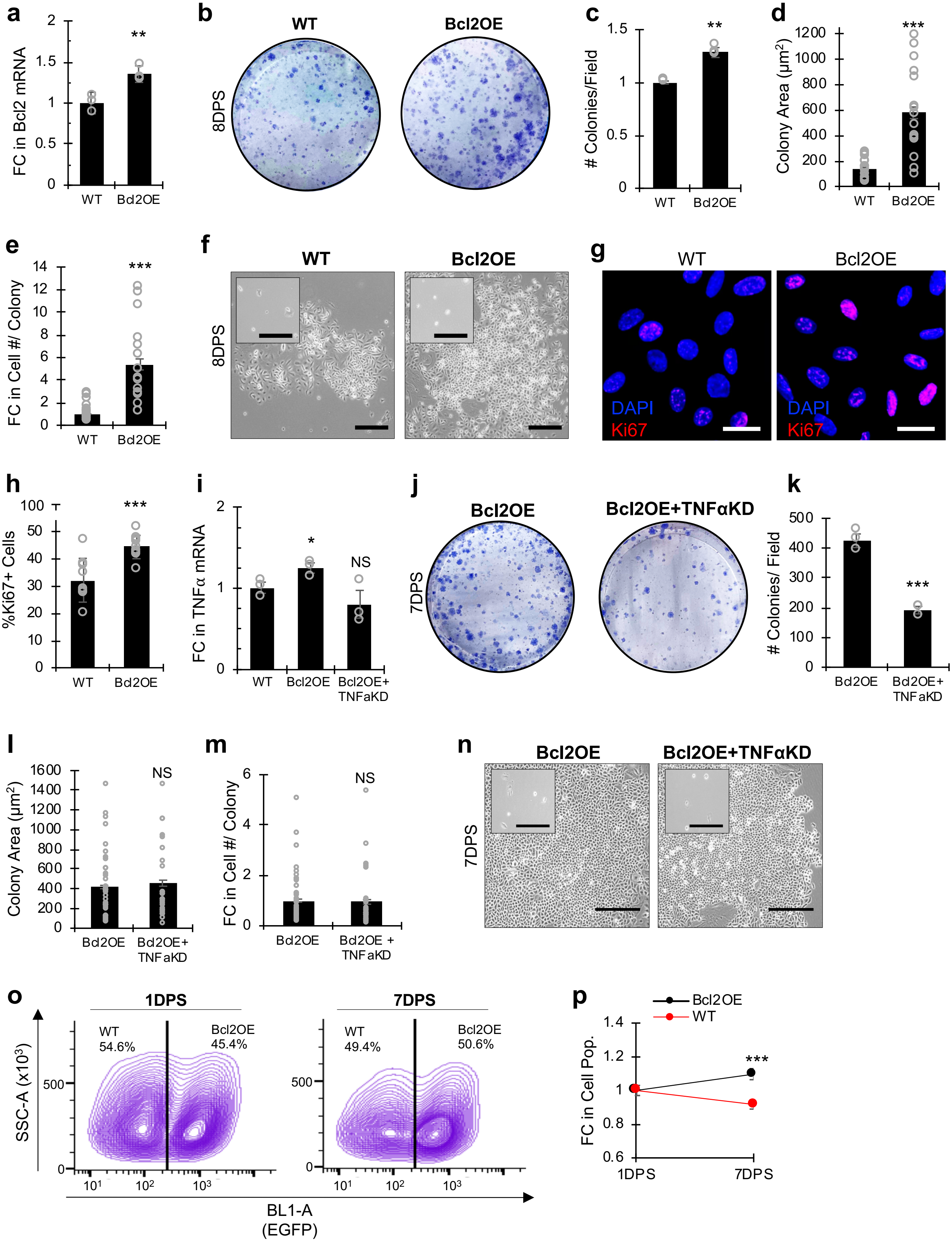

a
FC in Bcl2 mRNA
**
WT
Bcl2OE
b
WT
Bcl2OE
8DPS
c
Colonies/Field
**
d
Colony Area (µm²)
***
e
FC in Cell #/ Colony
***
f
8DPS
g
DAPI
Ki67
h
%Ki67+ Cells
***
i
FC in TNFα mRNA
*
NS
Bcl2OE+ TNFaKD
j
Bcl2OE
Bcl2OE+TNFαKD
7DPS
k
Colonies/ Field
***
l
Colony Area (µm²)
NS
m
FC in Cell #/ Colony
NS
n
Bcl2OE
Bcl2OE+TNFαKD
7DPS
o
1DPS
7DPS
WT 54.6%
Bcl2OE 45.4%
WT 49.4%
Bcl2OE 50.6%
SSC-A (x10³)
BL1-A (EGFP)
p
Bcl2OE
WT
FC in Cell Pop.
***
1DPS
7DPS

Supplementary Figure 5. Bcl2OE HFSCs exhibit enhanced proliferation and colony forming capacity that is attenuated with inhibition of TNFα.

a FC in mRNA levels of Bcl2 in Bcl2OE versus WT HFSCs.

b Toluidine Blue-O staining of WT (left) and Bcl2OE (right) HFSC colonies formed 8 days post-seeding (DPS) of colony formation assays.

c, d, e Quantification of the number of colonies per *n* = 3 fields (**c**), colony area (**d**) and average number of cells per colony (**e**) based on (**b**). A minimum of *n* = 15 colonies across *n* = 3 biological replicates were examined for (**d**) and (**e**).

f Brightfield images depicting WT (left) and Bcl2OE (right) HFSCs 8DPS and 1DPS (small inset).

g IF staining against Ki-67 in WT (left) and Bcl2OE (right) HFSCs.

h Quantification of percentage of Ki-67$^+$ cells based on (**g**). More than 3,800 cells across 9 randomly selected fields and *n* = 3 biological replicates examined per condition.

i FC in mRNA levels of TNFα in Bcl2OE and Bcl2OE+TNFaKD HFSCs versus WT controls.

j Toluidine Blue-O staining of Bcl2OE (left) and Bcl2OE+TNFαKD (right) HFSC colonies formed 7 days post-seeding (DPS).

k, l, m Quantification of the number of colonies per field (**k**), colony area (**l**), and average number of cells per colony (**m**) based on (**j**). A minimum of *n* = 25 colonies across *n* = 3 biological replicates examined.

n Brightfield images depicting Bcl2OE (left) and Bcl2OE+TNFαKD (right) HFSCs 7DPS and 1DPS (small inset).

o FACS plots depicting percentages of WT and Bcl2OE cell populations in co-culture 1 and 7DPS.

p Quantification of FC in population ratios from 1 to 7DPS pertaining to (**o**).

Scale bar represents 100μm for **f, n**; 25μm for **g**. *n* = 3 biological replicates per condition per experiment unless otherwise specified. Each experiment was repeated at least 2 times with similar results. All data are mean and ± SEM. P-values were determined by unpaired two-tailed t-test. P-value <0.05*, <0.01**, <0.001***.

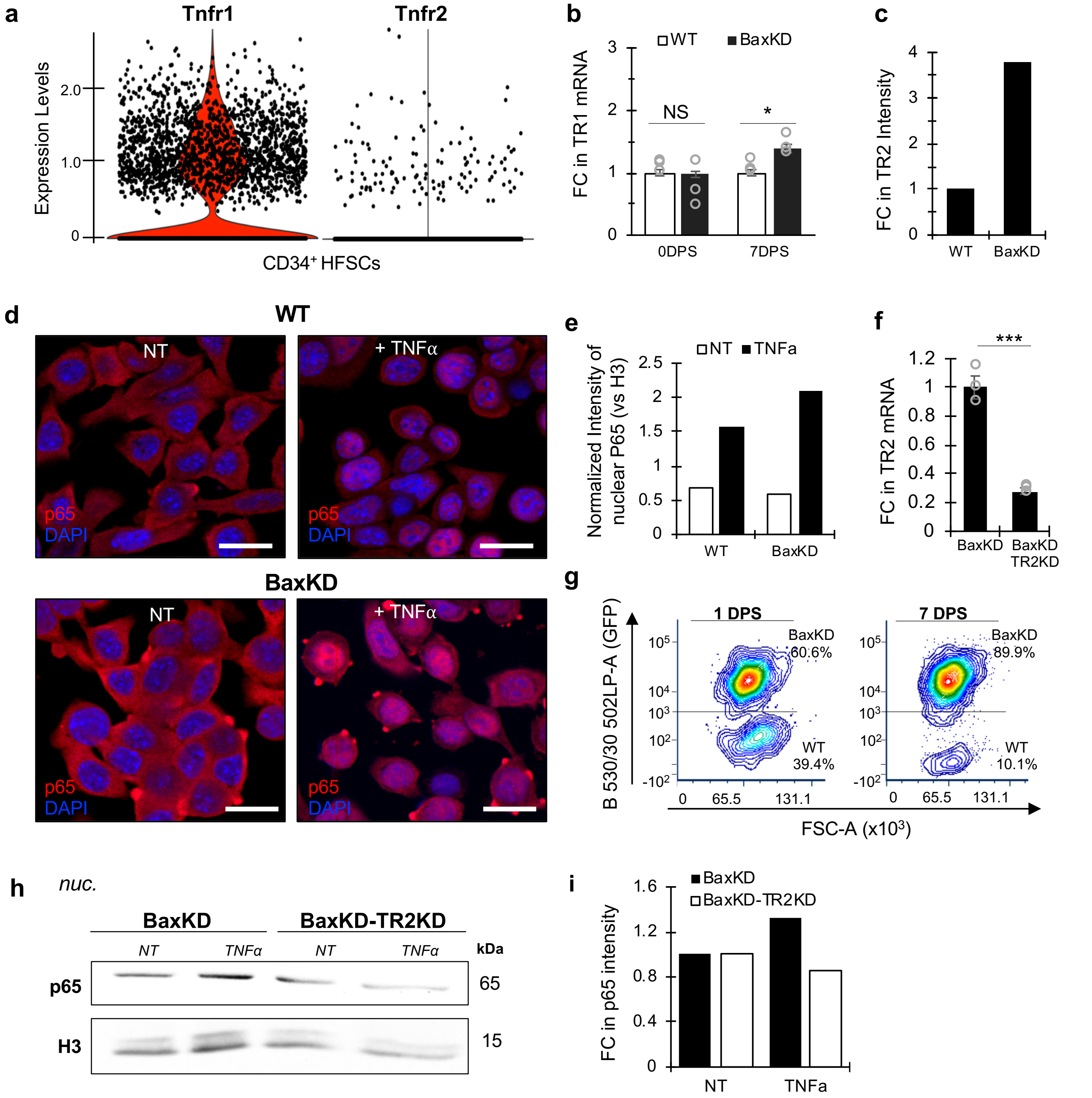

a
Tnfr1
Tnfr2
Expression Levels
2.0
1.0
0
CD34+ HFSCs
b
WT
BaxKD
FC in TR1 mRNA
NS
*
0DPS
7DPS
c
FC in TR2 Intensity
WT
BaxKD
d
WT
NT
+ TNFα
p65
DAPI
BaxKD
e
NT
TNFa
Normalized Intensity of nuclear P65 (vs H3)
WT
BaxKD
f
***
FC in TR2 mRNA
BaxKD
BaxKD TR2KD
g
1 DPS
7 DPS
B 530/30 502LP-A (GFP)
BaxKD 60.6%
WT 39.4%
BaxKD 89.9%
WT 10.1%
FSC-A (x10³)
h
nuc.
BaxKD
BaxKD-TR2KD
NT
TNFα
kDa
p65
65
H3
15
i
BaxKD
BaxKD-TR2KD
FC in p65 intensity
NT
TNFa

Supplementary Figure 6. Bax depleted cells differentially respond to TNFα stimulation.

a Violin plots from single-cell RNA-seq data depicting single cell distributions of Tnfr1 (left) and Tnfr2 (right) expression in the HFSC population. Each point represents an individual cell.

b FC in mRNA of Tnfr1 (TR1) in WT and BaxKD in co-culture 0 and 7DPS.

c FC in intensity of Tnfr2 (TR2) (normalized to GAPDH) based on Figure 5b.

d IF staining against p65 in WT and BaxKD HFSCs 5 min after TNFα treatment.

e FC in intensity of p65 (normalized to H3) based on Figure 5e.

f FC in mRNA levels of TR2 in BaxKD+TR2KD versus BaxKD HFSCs.

g FACS plot depicting percentage of BaxKD or WT HFSCs in co-cultures 1 and 7DPS representing control conditions corresponding to Fig. 5g-h.

h IB against p65 and H3 of nuclear fractions from BaxKD (left) or BaxKD+TR2KD (right) HFSCs after TNFα treatment.

i FC in intensity of p65 (normalized to H3) based on (**h**).

Scale bar represents 50μm in **d**. *n* = 3 biological replicates per condition per experiment unless otherwise specified. Each experiment was repeated at least 2 times with similar results. All data are mean and ± SEM. P-values were determined by unpaired two-tailed t-test. P-value <0.05*, <0.01**, <0.001***.

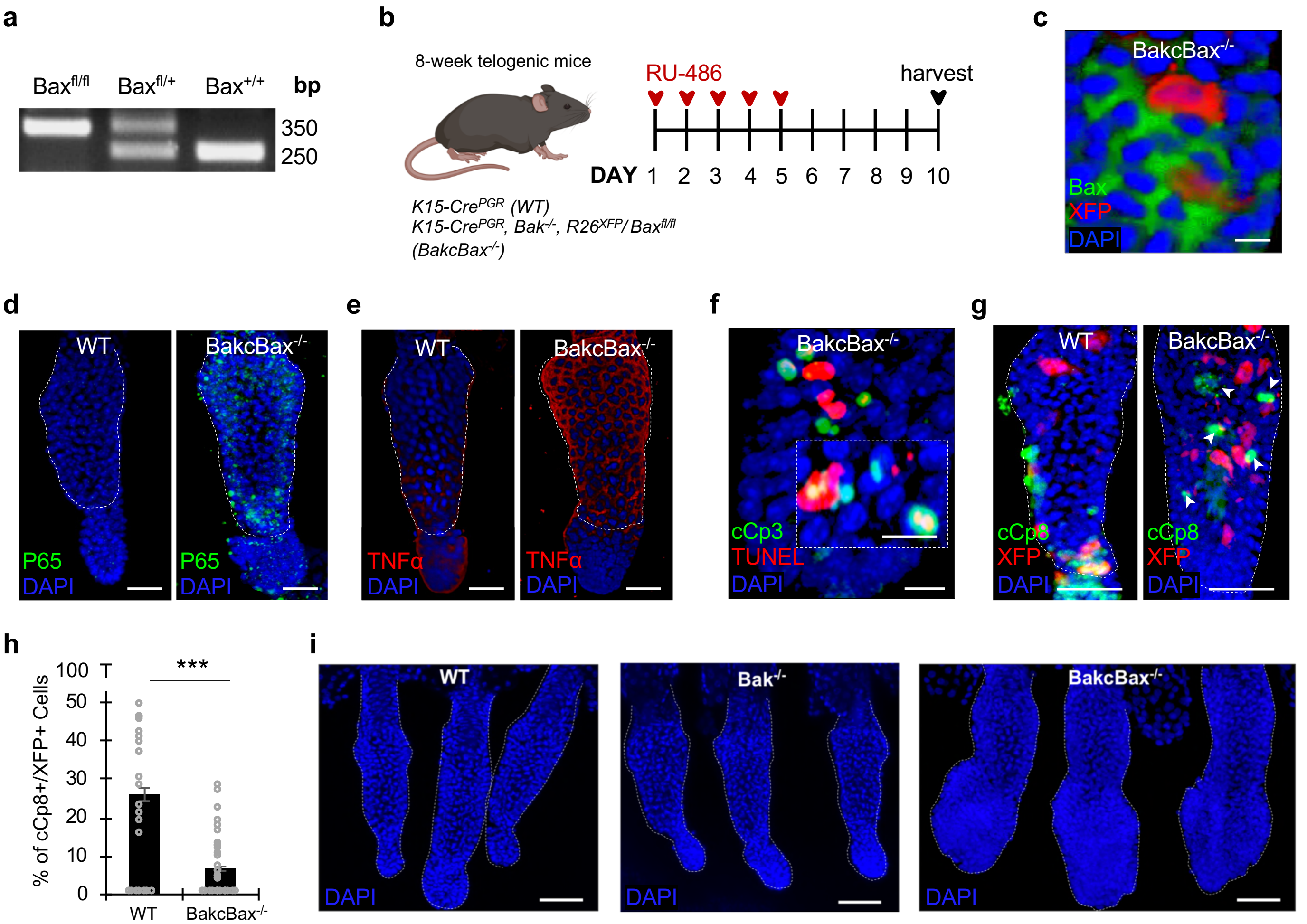
a
Baxfl/fl
Baxfl/+
Bax+/+
bp
350
250
b
8-week telogenic mice
RU-486
harvest
DAY 1 2 3 4 5 6 7 8 9 10
K15-CrePGR (WT)
K15-CrePGR, Bak-/-, R26XFP/Baxfl/fl
(BakcBax-/-)
c
BakcBax-/-
Bax
XFP
DAPI
d
WT
BakcBax-/-
P65
DAPI
e
WT
BakcBax-/-
TNFα
DAPI
f
BakcBax-/-
cCp3
TUNEL
DAPI
g
WT
BakcBax-/-
cCp8
XFP
DAPI
h
% of cCp8+/XFP+ Cells
100
50
40
30
20
10
0
***
WT
BakcBax-/-
i
WT
Bak-/-
BakcBax-/-
DAPI

Supplementary Figure 7. Bax depletion results in elevated p65 and TNFα levels, and correlates with non-cell autonomous apoptotic induction and tissue hypertrophy *in vivo*.

a Nucleic acid gel depicting genotyping for floxed (fl/fl), heterozygous floxed (fl/+), or WT (+/+) Bax allele.

b Schematic depiction of timeline for induction of *BakcBax*$^{-/-}$ mice with RU486.

c IF staining against Bax in HFs from tail epidermis whole mounts (TWMs).

d, e IF staining against p65 (**d**) and TNFα (**e**) in HFs from WT and *BakcBax*$^{-/-}$ TWMs. Dotted lines mark the HFSC bulge region.

f IF staining against cCp3 and TUNEL assay labeling in a HF from *BakcBax*$^{-/-}$ TWMs.

g IF staining against cCp8 in WT and *BakcBax*$^{-/-}$ TWMs. Arrowheads denote cCp8^{+} cells adjacent to induced XFP (red) cells in *BakcBax*$^{-/-}$ TWMs. Dotted lines mark the HFSC bulge region.

h Percentage of cCp8^{+}XFP^{+} (double positive) cells in WT and *BakcBax*$^{-/-}$ HFs based on (**g**). *n* = 3 biological replicates for both WT and *BakcBax*$^{-/-}$ with a minimum of 25 HFs examined per condition.

i DAPI labeling of WT (left), *Bak*$^{-/-}$ (middle), and *BakcBax*$^{-/-}$ (right) HFs from TWMs. Dotted lines mark the HF boundary.

Scale bar represents 10μm in **c**; 25μm in **d, e**; 20μm in **f**; 100μm in **g**; 50μm **i**. *n* = 3 biological replicates per condition per experiment unless otherwise specified. Each experiment was repeated at least 2 times with similar results. All data are mean and ± SEM. P-values were determined by unpaired two-tailed t-test. P-value <0.05*, <0.01**, <0.001***.

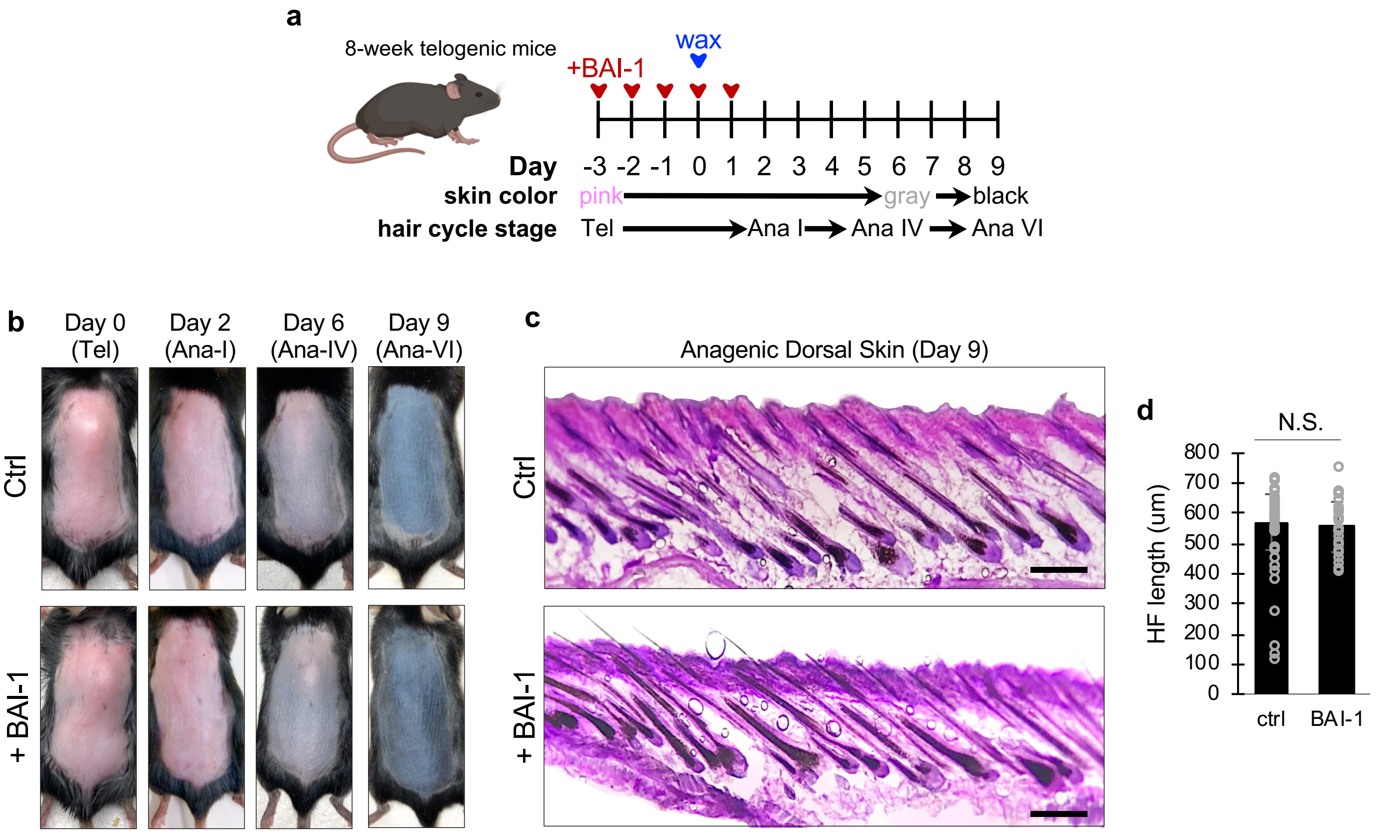
a
8-week telogenic mice
+BAI-1
wax
Day
-3 -2 -1 0 1 2 3 4 5 6 7 8 9
skin color
pink
gray
black
hair cycle stage
Tel
Ana I
Ana IV
Ana VI
b
Day 0 (Tel)
Day 2 (Ana-I)
Day 6 (Ana-IV)
Day 9 (Ana-VI)
Ctrl
+ BAI-1
c
Anagenic Dorsal Skin (Day 9)
Ctrl
+ BAI-1
d
N.S.
HF length (um)
0
100
200
300
400
500
600
700
800
ctrl
BAI-1

Supplementary Figure 8. Bax inhibition does not show alteration of the HF cycle.

a Schematic depiction of depilation experiments with BAI-1 (Bax inhibitor).

b Representative images of BAI-1 or vehicle (NT) treated mice 0, 2, 6, and 9 days post-depilation.

c H&E staining of BAI-1 or NT treated dorsal skin sections 9 days post-depilation.

d Quantification of HF length based on (**c**). More than 25 HFs across *n* = 3 biological replicates were evaluated per condition.

Scale bar represents 200μm in **c**. *n* = 3 biological replicates per condition per experiment unless otherwise specified. All data are mean and ± SEM. P-values were determined by unpaired two-tailed t-test. P-value <0.05*, <0.01**, <0.001***.

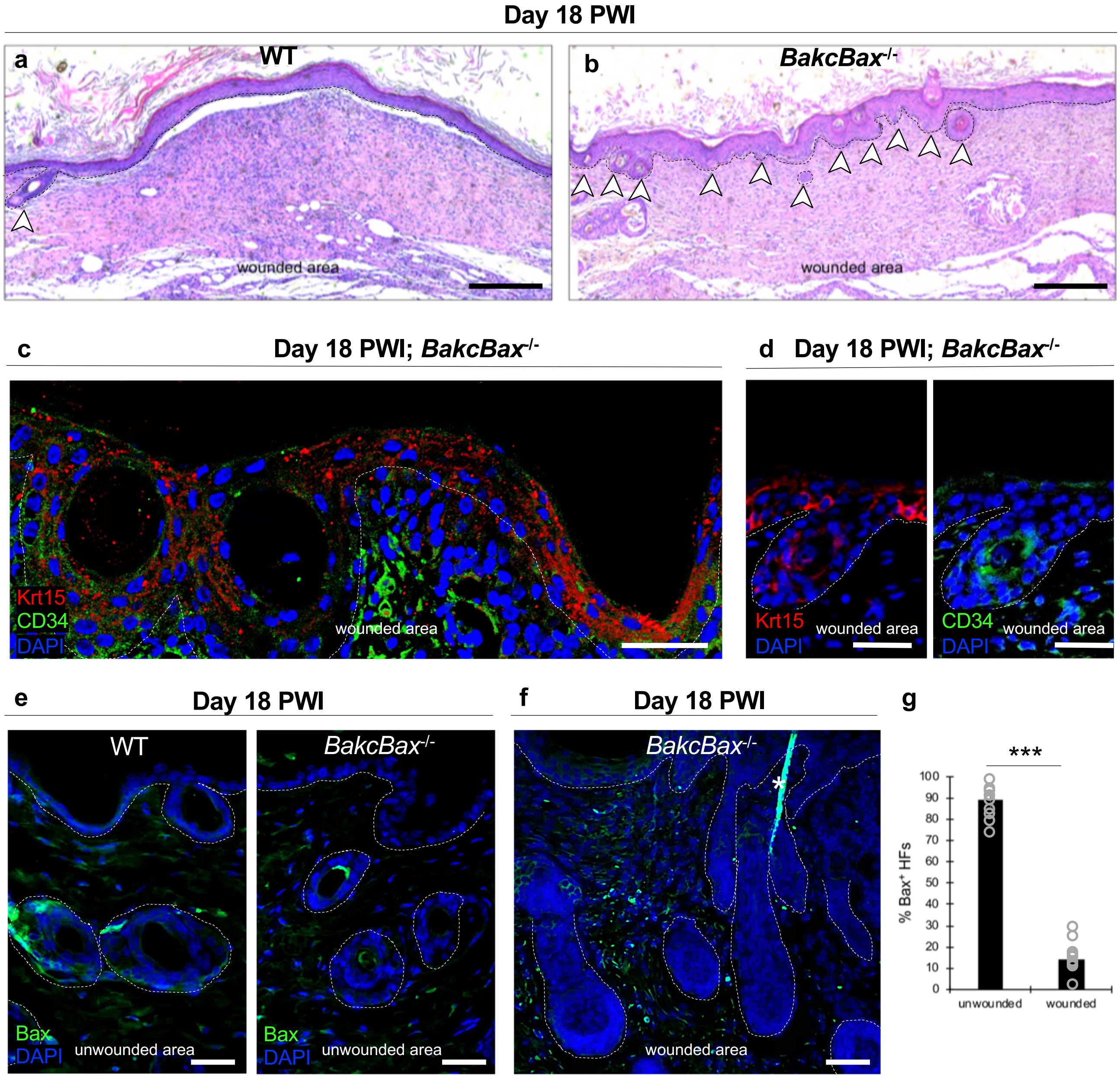
Day 18 PWI
a
WT
wounded area
b
BakcBax-/-
wounded area
c
Day 18 PWI; BakcBax-/-
Krt15
CD34
DAPI
wounded area
d
Day 18 PWI; BakcBax-/-
Krt15
DAPI
wounded area
CD34
DAPI
wounded area
e
Day 18 PWI
WT
BakcBax-/-
Bax
DAPI
unwounded area
Bax
DAPI
unwounded area
f
Day 18 PWI
BakcBax-/-
*
wounded area
g
***
100
90
80
70
60
50
40
30
20
10
0
% Bax+ HFs
unwounded
wounded

Supplementary Figure 9. Bax depletion results in accelerated wound healing and formation of *de novo* hair follicles *in vivo*.

a, b H&E staining of WT and *BakcBax*$^{-/-}$ dorsal skin sections from within the wound bed 18 days post wound induction (PWI). Dashed lines denote epidermal borders and arrowheads denote formation of *de novo* HFs.

c, d IF staining against CD34 (**c**) and Krt15 (**d**) from within the wound bed of *BakcBax*$^{-/-}$ dorsal skin sections 18 days PWI. Dashed lines denote epidermal boundaries.

e IF staining against Bax in WT and *BakcBax*$^{-/-}$ dorsal skin sections from unwounded areas adjacent to the wound bed 18 days PWI. Dashed lines denote epidermal-dermal border.

f IF staining against Bax of regenerated HFs in *BakcBax*$^{-/-}$ dorsal skin section from within the wound bed 18 days PWI. Dashed lines denote epidermal-dermal border. White asterisk denotes autofluorescence.

g Percentage of HFs expressing Bax in unwounded versus wounded regions from *BakcBax*$^{-/-}$ dorsal skin sections 18 days PWI based on (**e; right panel, f**).

Scale bar represents 150μm in **a, b**; 100μm in **c, d**; 25μm in **e**; 100μm in **f**. *n* = 3 biological replicates per condition per experiment unless otherwise specified. All data are mean and ± SEM. P-values were determined by unpaired two-tailed t-test. P-value <0.05*, <0.01**, <0.001***.

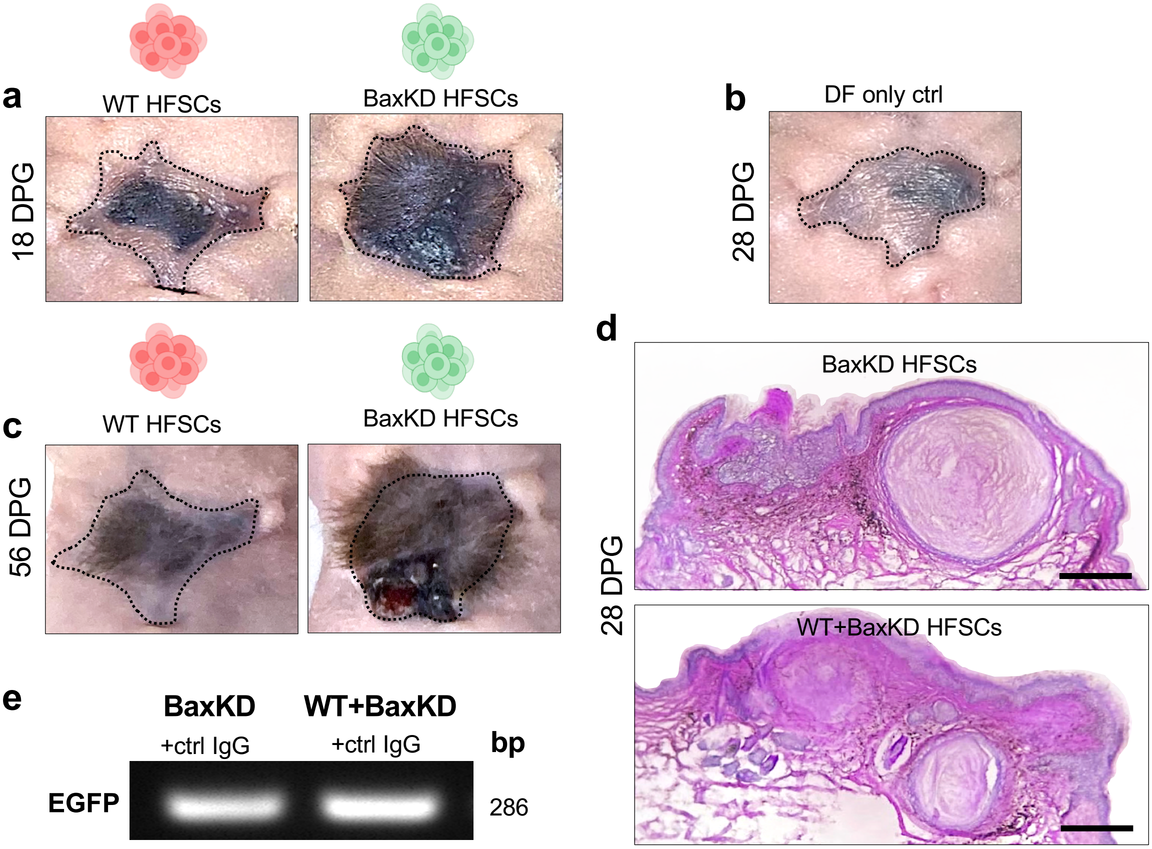
a
WT HFSCs
BaxKD HFSCs
18 DPG
b
DF only ctrl
28 DPG
c
WT HFSCs
BaxKD HFSCs
56 DPG
d
BaxKD HFSCs
WT+BaxKD HFSCs
28 DPG
e
BaxKD
+ctrl IgG
WT+BaxKD
+ctrl IgG
bp
EGFP
286

Supplementary Figure 10. Transplanted BaxKD HFSCs exhibit enhanced HF generation capacity and development of aberrant growths *in vivo*.

a Images representing hair growth in grafts derived from WT (left) or BaxKD (right) HFSCs after 18 days post grafting (DPG).

b Image representing lack of hair growth in control graft derived from neonatal DFs only after 28 DPG.

c Images representing hair growth in grafts derived from WT (left) or BaxKD (right) HFSCs after 56 DPG.

d H&E staining of graft sections including regions of hyperplastic growths from BaxKD only (top) or WT+BaxKD (bottom) HFSCs 28 DPG.

e Nucleic acid gel depicting EGFP amplicons from gDNA of grafts derived from BaxKD (left lane) or mixed population (middle and right lanes) HFSCs.

Scale bar represents 100μm in **d**. *n* = 3 biological replicates per condition per experiment unless otherwise specified. Each experiment was repeated at least 2 times with similar results.

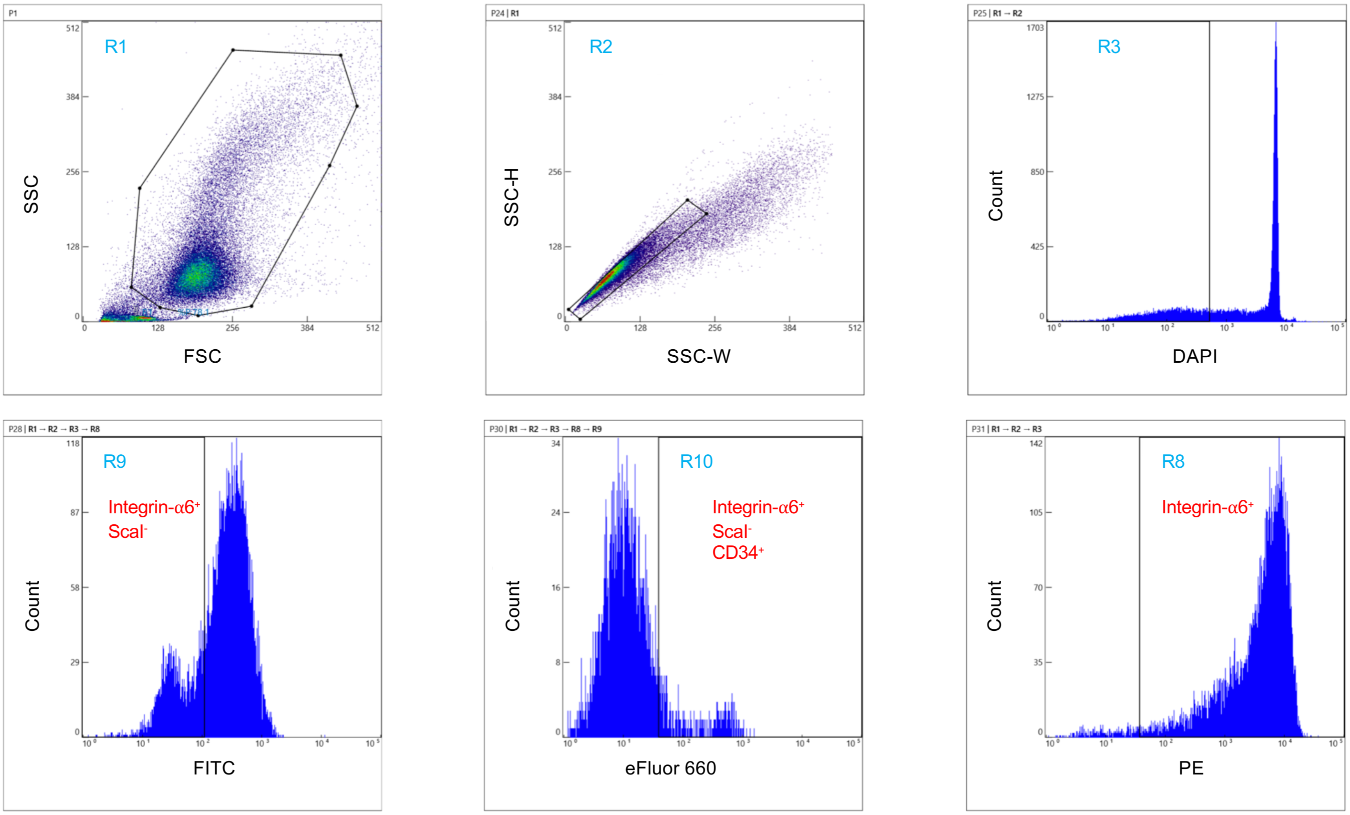

Supplementary Figure 11. Isolation of mouse hair follicle stem cells (HFSCs).

FACS strategy for the isolation of Integrin-$\alpha 6^+$ ScaI$^-$CD34$^+$ HFSCs from mouse skin epidermal cell populations.

Source Data (Supplementary Figures)

SUPP. FIG 1C

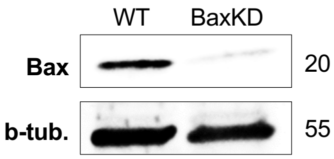

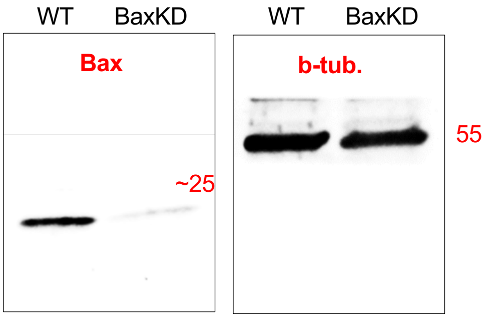

SUPP. FIG 4J

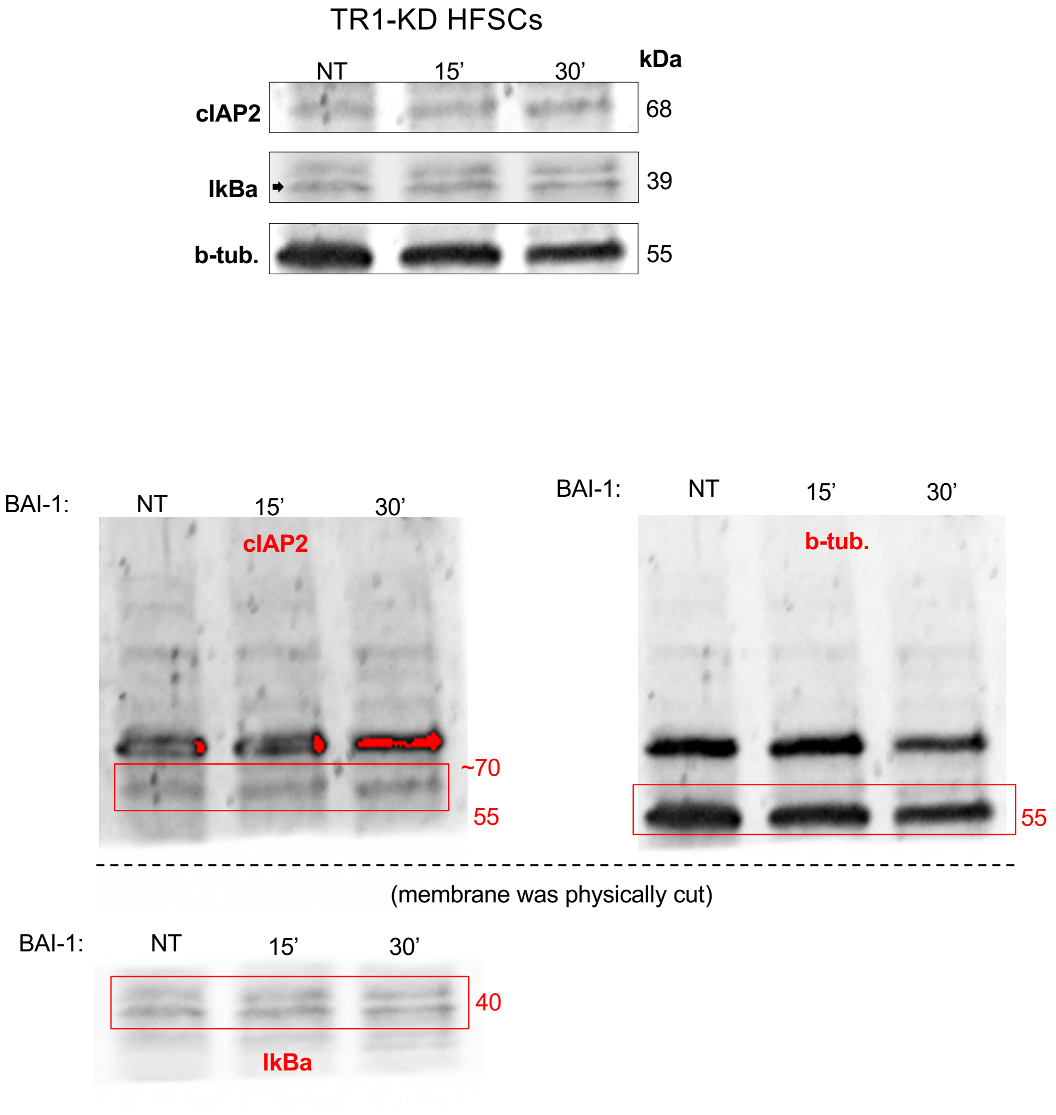
TR1-KD HFSCs
NT
15’
30’
kDa
cIAP2
68
IkBa
39
b-tub.
55
BAI-1:
NT
15’
30’
cIAP2
~70
55
BAI-1:
NT
15’
30’
b-tub.
55
(membrane was physically cut)
BAI-1:
NT
15’
30’
40
IkBa

SUPP. FIG 4L

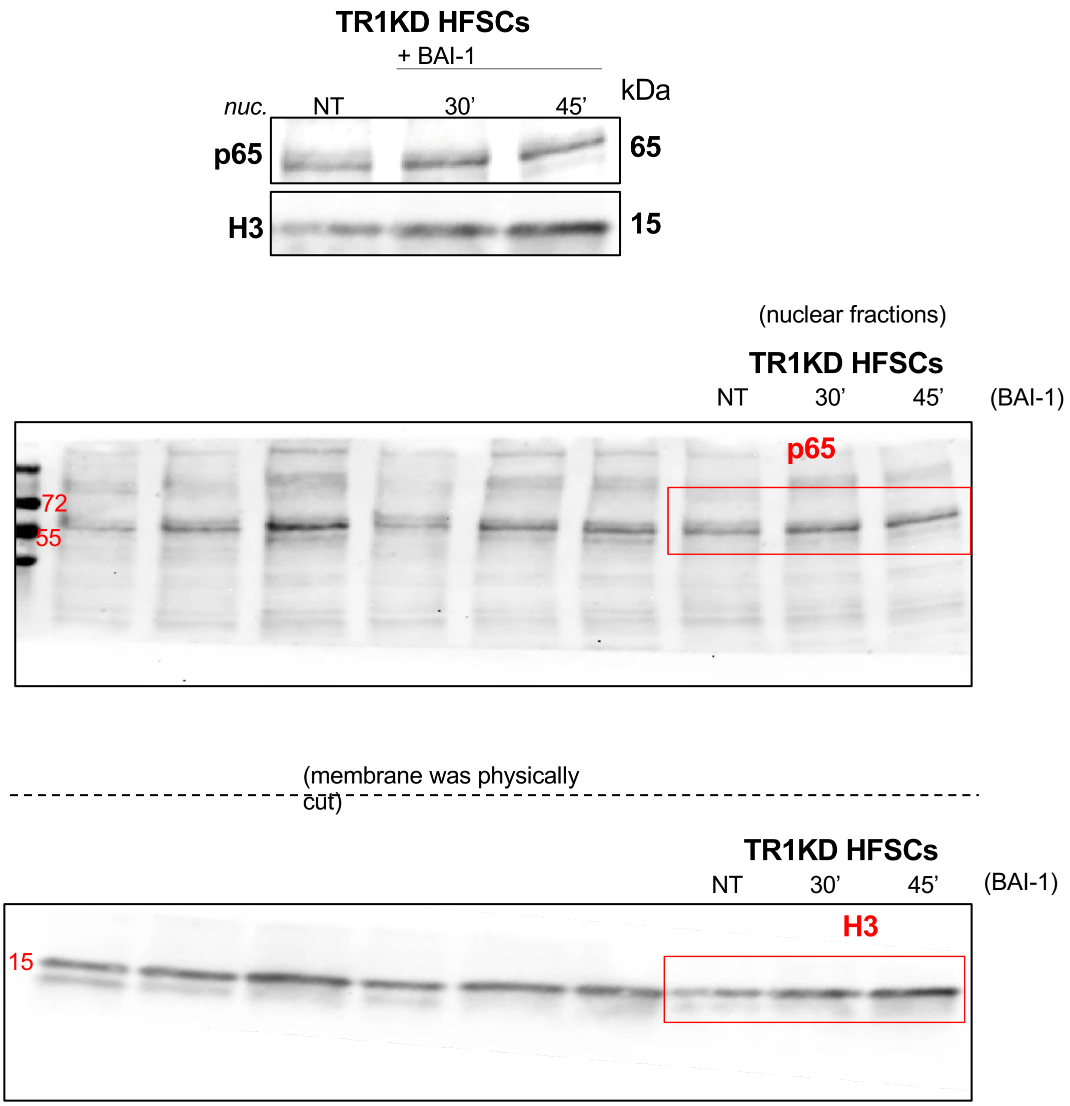
TR1KD HFSCs
+ BAI-1
nuc.
NT
30’
45’
kDa
p65
65
H3
15
(nuclear fractions)
TR1KD HFSCs
NT
30’
45’
(BAI-1)
p65
72
55
(membrane was physically cut)
TR1KD HFSCs
NT
30’
45’
(BAI-1)
H3
15

SUPP. FIG 6H

nuclear fractions from HFSCs

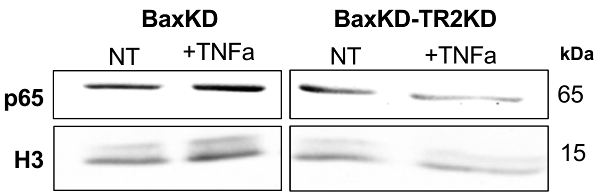

*In the figure panel, lanes are separated by boxes for visual clarity

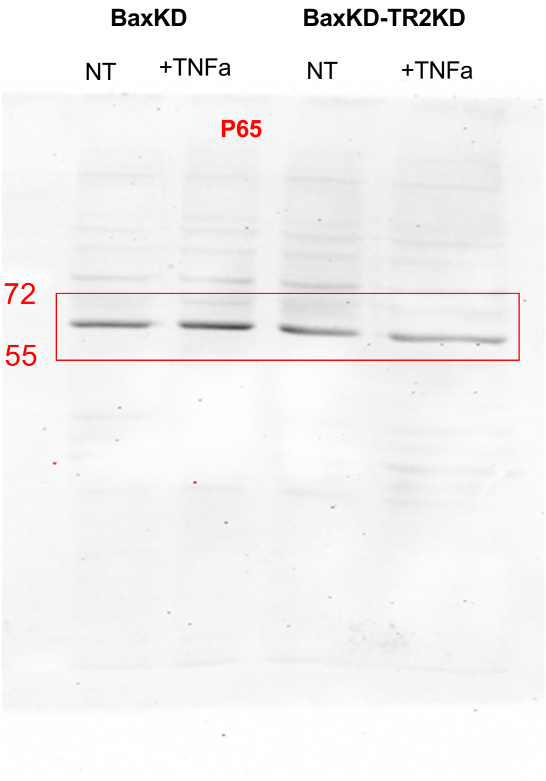

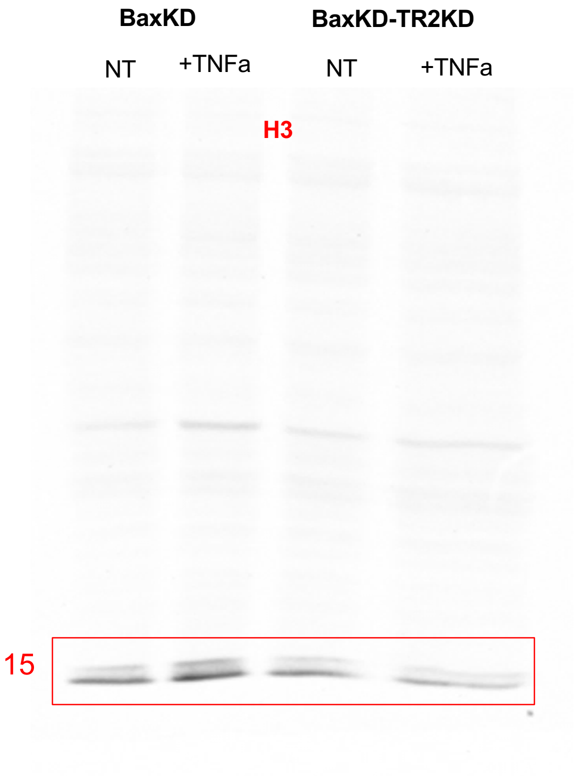

SUPP. FIG 7A

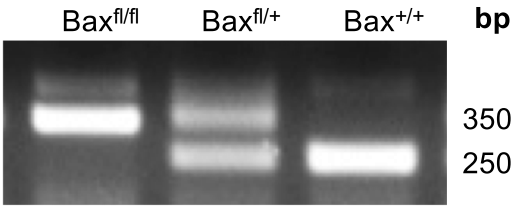

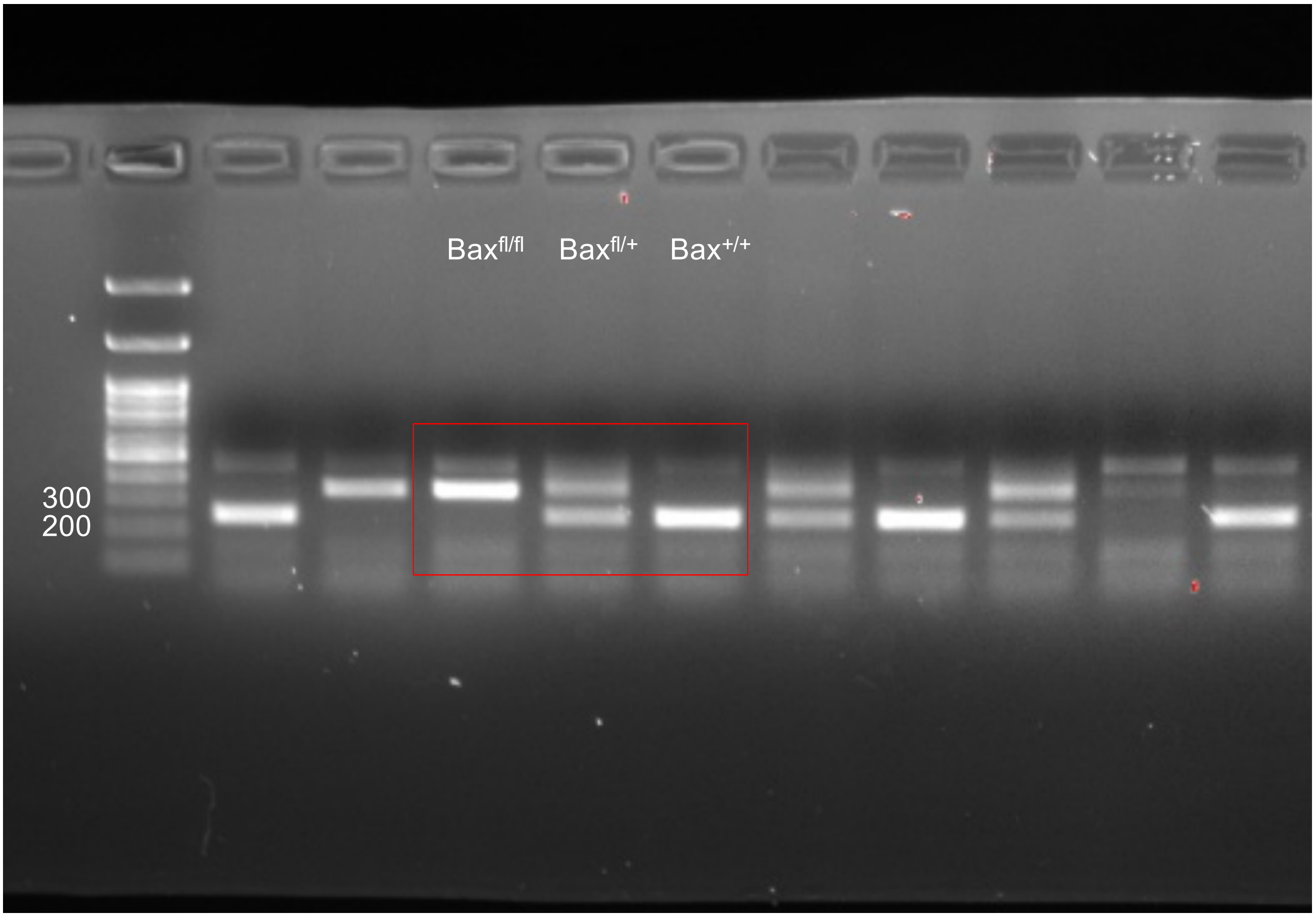

SUPP. FIG. 10E

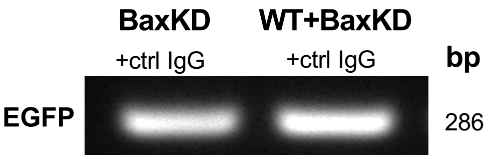
BaxKD
WT+BaxKD
+ctrl IgG
+ctrl IgG
bp
EGFP
286

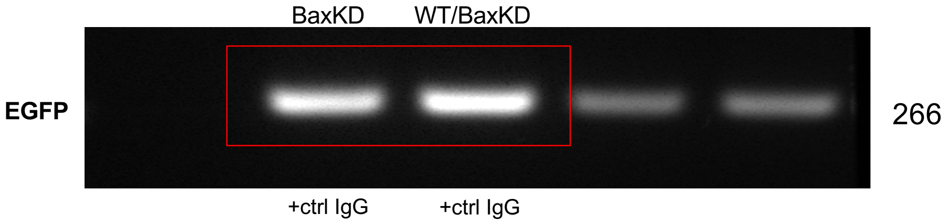
BaxKD
WT/BaxKD
EGFP
266
+ctrl IgG
+ctrl IgG
