# Supplementary material for: Apoptotic dysregulation mediates stem cell competition and tissue regeneration
Source: Nat Commun. 2023 Nov 20;14:7547. doi: 10.1038/s41467-023-41684-x (PMC10662150; doi:10.1038/s41467-023-41684-x)
Supplement: Supplementary file 2 — Description of Additional Supplementary Files [file 41467_2023_41684_MOESM2_ESM.pdf]

Description of Additional Supplementary Files

Supplementary Movie 1. WT (depicted in blue) and BaxKD (depicted in red) hair follicle stem cell (HFSC) co-cultures 5 days post seeding (DPS) under homeostatic culture conditions over the course of 15 hours and various imaging fields.

Supplementary Movie 2. WT (depicted in blue) and BaxKD (depicted in red) hair follicle stem cell (HFSC) co-cultures 5 days post seeding (DPS) immediately upon stimulation with TNF-alpha (20ng/mL) over the course of 15 hours and various imaging fields.
